# Identification of Phage RNA Polymerases That Minimize Double-Stranded RNA By-Product Formation and Their Characterization via In Vitro Transcription

**DOI:** 10.3390/microorganisms14030564

**Published:** 2026-03-02

**Authors:** Lilian Göldel, Carsten Bornhövd, Johannes Kabisch, Aron Eiermann, Joseph Heenan, Thomas Brück, Hagen Richter

**Affiliations:** 1 Wacker Chemie AG, 81379 Munich, Germany; lilian.goeldel@wacker.com (L.G.); carsten.bornhoevd@wacker.com (C.B.); 2 P2P Bio GmbH, 64293 Darmstadt, Germany; johannes.kabisch@ntnu.no (J.K.); mail@aroneiermann.de (A.E.); 3 Department of Biotechnology and Food Science, NTNU Norwegian University of Science and Technology, 7491 Trondheim, Norway; 4 Proteineer GmbH, 63128 Dietzenbach, Germany; joe@proteineer.com; 5 Werner Siemens-Chair of Synthetic Biotechnology, TUM School of Natural Sciences, Technical University of Munich (TUM), 85748 Garching, Germany

**Keywords:** viral RNA polymerase, in vitro transcription, dsRNA, mRNA manufacturing, T7 RNA polymerase, genome mining, promoter screening, RNA aptamer

## Abstract

Therapeutics based on RNA are commonly produced via biocatalytic approaches using RNA polymerases. The most frequently applied enzyme is the RNA polymerase of Enterobacteria phage T7. However, this enzyme has unfavorable properties, like the formation of double-stranded RNA (dsRNA). This undesired by-product can activate the innate immune system via pattern recognition receptors and cause inflammation. Removal of the contaminant is time-consuming and expensive. In this work, we applied a genome mining approach to identify unidentified single-subunit RNA polymerases with minimal dsRNA generation. A large meta database was screened, and 74 sequences were selected. Two RNA polymerases generating barely detectable amounts of dsRNA were identified from the initial sequence portfolio. Their promoters were detected via a fluorescent RNA aptamer screening, and slightly acidic transcription conditions were established. Further activity characterization showed a significant reduction of dsRNA to 0.001% and 0.02%. Due to these beneficial attributes, these RNA polymerases generate mRNA with enhanced stability, which most likely lowers the immune response towards the desired mRNA. This could be especially useful for producing long RNAs, such as self-amplifying RNA, as these typically require improved stability and low dsRNA content.

## 1. Introduction

The success of mRNA-based COVID-19 vaccines, such as Comirnaty or Spikevax has led to intensified research to develop other nucleic acid-based therapeutics and vaccines [1,2,3,4]. Due to its higher stability compared to mRNA, plasmid DNA (pDNA) was the main area of nucleic acid vaccine research. However, plasmid DNA-based vaccines have drawbacks like the potential threat of genome integration and mutagenesis. Further, plasmid DNA must enter the nucleus to be effective, which requires dividing cells, thereby increasing the technical demand for this genomic approach.

In contrast, mRNA therapeutics do not need to enter the nucleus, thereby eliminating immunological risks and the difficulties in target sequence delivery [4,5]. The current process for pharmaceutical long RNA production is the in vitro run-off transcription (IVT) [4,6]. In this enzymatically driven reaction, single-stranded RNA is generated using nucleoside triphosphates (NTPs) complementary to a defined DNA template. The template encodes the promoter of an RNA polymerase, commonly the T7 RNA polymerase, in conjunction with a sequence of interest. In the initiation phase, the T7 RNA polymerase recognizes and binds to its specific promoter region and dissociates the template from the non-template DNA strand, thereby forming an unstable initiation complex (IC). Promoter recognition occurs via the N-terminal domain (NTD) and the specificity loop of the polymerase [7]. Upon formation of the initiation complex, the template strand is guided to the active site [8,9,10]. Here, seven amino acids orchestrate catalysis. Specifically, Lys-627, Lys-631, Met-635, and Tyr-639 enable substrate and nucleoside triphosphate (NTP) binding, while Asp-537 and Asp-812 coordinate two Mg2+ ions to contribute to the formation of the phosphodiester bond between NTP and the already synthesized RNA [8]. Ultimately, Lys-472 is suggested to assist in the release of the by-product pyrophosphate [11]. In this state, many short RNAs (less than 9 nt) can be released from the complex, followed by a new initiation of RNA synthesis by the RNA polymerase, which is still bound to the promoter region of the DNA. This process is called abortive cycling [8,12]. When a length of more than nine nucleotides is exceeded, the promoter is released by the RNA polymerase and the unstable initiation complex transitions into the stable elongation complex (EC). This complex does not show the same abortive properties but instead is characterized by a highly processive nature, which can be stopped via termination sequences or the end of a linear DNA (“run-off” transcription) [12,13,14].

While several different RNA polymerases are characterized and commercially available, e.g., from Enterobacteria phage T3 or Salmonella phage SP6 [15,16,17,18], the RNA polymerase of Enterobacteria phage T7 is the most commonly used in RNA manufacturing. Its high fidelity, processivity, and product yields make T7 RNA polymerase variants attractive for laboratory and industrial settings [12,19,20]. While being a very efficient enzyme, this RNA polymerase has certain disadvantages, such as tendency to abortive cycling, 3′-end extension, and promoter-independent RNA synthesis [21,22,23]. Abortive cycling is a repetitive step at the beginning of transcription, generating short RNA species at a high rate. These transcripts can bind to the full-length RNA. Since this RNA polymerase exhibits unusual RNA-dependent activity, it can use the hybridized transcripts as primer and extend the RNA, resulting in dsRNA [22,24]. Furthermore, it was observed that some bacteriophage-derived RNA polymerases, including T7, are capable of transcribing RNA from DNA lacking a corresponding promoter. This implies that the polymerase is able to change at the end of the DNA from the template strand to the non-template and generate antisense RNA, which can form dsRNA in the presence of its sense RNA [25,26]. Moreover, T7 RNA polymerase can use the 3′-end of its product as template and extend it, thereby creating dsRNA. This synthesis is driven by the 3′-end of full-length RNA forming a dsRNA hairpin with itself, to which the polymerase can bind [27]. The precise reactions mechanisms remain elusive [26]. These by-products are inconvenient for manufacturing as they reduce yield and mRNA integrity, increase associated costs, and trigger an unwanted immune response [26,28].

Inside the cell, several pattern recognition receptors, as part of the innate immune system, detect dsRNA. Cytosolic RIG-I-like receptors RIG-I and melanoma differentiation-associated protein 5 recognize dsRNA and activate the mitochondrial antiviral-signaling pathway, inducing the release of proinflammatory cytokines. The NOD-like receptors NLRP1 and NLRP3 bind dsRNA and can cause the secretion of cytokines IL-1β and IL-18. Additionally, the RNA itself exhibits an immunogenic character, as it may resemble viral genomes [29].

To minimize unintended immune reactions, naturally occurring modified NTPs instead of unmodified NTPs were used, as they apparently lower the immunostimulatory potential [30]. RNA derived from mammalian cells, e.g., ribosomal RNA, is heavily modified. Conversely, prokaryotic RNA has little to no modifications [31,32]. Therefore, the incorporation of certain modifications assists in lowering the cellular immune response. Further strategies for reduced immunogenicity focus on reducing the amount of by-products, by chromatography, for example [33,34]. While this method is very precise, it comes at the cost of yield and efficiency, making it difficult and expensive. Avoiding by-product formation, these later bottlenecks for drug development can be omitted. One approach is the use of engineered RNA polymerases [12,35]. The T7 mutant P266L produces less abortive RNA species compared to the wild-type species, as this mutation destabilizes promoter interactions while stabilizing the elongation complex. Furthermore, a major decrease in dsRNA production was accomplished by introducing a double mutation (G47A + 884G), which favors the transition from the initiation to the elongation complex. Alternatively, it is possible to identify not-characterized RNA polymerases with fewer by-products forming properties.

In this study, a sequence data-driven genome mining approach was applied, which used a metagenomic database to detect unidentified RNA polymerase sequences. After rational filtering of approximately 2700 genes, 74 hits were selected. Subsequently, two candidates showed good activity, and via a fluorescent RNA aptamer screening, optimal promoter sequences were identified. Essential IVT parameters, such as pH value, were investigated for both enzymes, showing an optimal pH of 6.5. The dsRNA content generated during in vitro transcriptions with the newly identified RNA polymerases was greatly reduced compared to the standard T7 RNA polymerase. The two identified enzymes in this work extend the toolbox of RNA manufacturing and, thereby, help address the challenges linked to the quality of future novel and improved RNA-based vaccines.

## 2. Materials and Methods

The experiments described in this study were conducted using a range of commercially available materials and kits, which are summarized in Table 1.

### 2.1. Creation of an RNA Polymerase (RNAP) Variant Library and Gene Synthesis

For the identification of not-characterized RNA polymerase genes, the GeneStore algorithm of Proteineer GmbH (Dietzenbach, Germany), which incorporates a wide range of metagenomic data sources, was used to help identify relevant proteins. Major components of the GeneStore are derived from sources such as the European Nucleotide Archive [36] and the Joint Genome Institute Genome Portal [37], as well as proprietary metagenomic data sources. Additionally, sequences from sources such as Blackwell et al. [38] and the Serratus collection of Edgar et al. [39] were searched. When no assembly was already available in the GeneStore, the MEGAHIT de novo assembly tool was utilized to assemble nucleotide collections [40]. Contigs were selected for further analysis based on having nucleotide sequences that returned a significant hit via the TBLASTN search algorithm with a default e-value cutoff to one of three reference query proteins—either the T7, SP6, or T3 phage single-subunit DNA-dependent RNA polymerase. For contigs matching one of the three reference queries, the following algorithm was run: First, annotation of assemblies was performed using Prokka [41]. Sequences annotated as containing both a capsid protein and a single-subunit viral polymerase were retained for further analysis. Second, DIAMOND [42] was used to filter for hit novelty. Here, novelty was defined as the absence of a 100% match to any protein sequence from a commonly available source, such as the GenBank/NR database. This approach enriched the results for unidentified metagenomic hits. Third, hits were ranked into blocks based on sequence identity to the reference protein (30–80%). The presence of a promoter for the capsid protein, amino acid length greater than 600, was required, and hits were selected from the top blocks. Modeling of the protein structure of these hits was performed by the artificial intelligence (AI) program AlphaFold [43,44]. The resulting gene sequences were ordered in pET-29b(+) expression plasmids from Twist Bioscience.

### 2.2. Protein Expression and Purification

Plasmid DNA containing the genes for not-characterized RNA polymerases (including N-terminal His6-tag) was transformed into *E. coli* BL21 (DE3) employing the heat shock approach. A seed culture was prepared by inoculation of 5 mL LB medium containing 50 µg/mL kanamycin (Sigma Aldrich, Burlington,, MA, USA), which was incubated at 37 °C overnight. Using the seed culture, 200 mL LB medium containing 50 µg/mL kanamycin was inoculated to an OD600 = 0.1 and incubated at 37 °C. At an OD600 of ~0.6, 0.5 M IPTG (VWR) was added to induce protein production. After 6 h, cells were harvested via centrifugation (8800× *g*, 5 min, 25 °C). The pellet was resuspended in lysis buffer (50 mM Tris, 100 mM NaCl, 5% glycerol, pH 8 for No. 2049 or pH 8.5 for No. 1575) and incubated with lysozyme (ThermoFisher, Waltham, MA, USA) on ice for 30 min. Cells were lysed via ultrasonication (Branson, Brookfield, CT, USA). After centrifugation (10,000× *g*, 30 min, 4 °C) and sterile filtration (⌀ = 0.2 µm), the lysate was purified via gravity flow affinity chromatography (Ni-NTA, Qiagen). After loading 5 mL lysate, the column was washed with 10 mL wash buffer (50 mM Tris, 100 mM NaCl, 5% glycerol, 20 mM imidazole). Elution was performed with 2.5 mL elution buffer (50 mM Tris, 100 mM NaCl, 5% glycerol, 500 mM imidazole).

Alternatively, proteins were purified via fast protein liquid chromatography (FPLC), where the filtered lysate was loaded on a 1 mL HisTrap FF (Cytiva, Marlborough, MA, USA) column at a flow rate of 2 mL/min. After washing (50 mM Tris, 100 mM NaCl, 5% glycerol, pH 8 for No. 2049 or pH 8.5 for No. 1575), proteins were eluted with an imidazole gradient (0–500 mM). After analysis via SDS-PAGE, fractions with newly identified RNAPs were identified, pooled, and mixed 1:1 with low salt wash buffer (50 mM Tris, 5% glycerol, pH 8 for No. 2049 or pH 8.5 for No. 1575). The mixture was loaded on a 5 mM HiTrap Q (Cytiva) at a flow rate of 2 mL/min. The column was washed with low salt wash buffer, and protein was eluted with a NaCl gradient (0–1 M) using a high salt wash buffer (50 mM Tris, 1 M NaCl, 5% glycerol, pH 8 for No. 2049 or pH 8.5 for No. 1575). After analysis via SDS-PAGE, fractions with newly characterized proteins were identified, pooled, and the proteins were transferred into storage buffer (50 mM Tris, 100 mM NaCl, 50% glycerol, pH 8 for No. 2049 or pH 8.5 for No. 1575) using a PD-10 desalting column (Cytiva). The purity was determined via SDS-PAGE (Gel Doc XR+ documentation system and ImageLab (BioRad, Hercules, CA, USA)), and the protein solution was stored at −20 °C.

### 2.3. Promoter Library Creation and DNA Template Production

For each potential RNA polymerase, the region 100 bp upstream of a gene coding for a predicted capsid protein was selected as a potential promoter region. Additionally, the genomic data sets of proteins No. 1575, 2037, 2038, 2039, 2040, 2045, 2049, and 2051 were screened for the phage promoter core sequence (bac core region of T3, T7, and SP6 phage promoters) [10,45]. Matching regions, which were up to 150 bp upstream of a predicted gene and not part of a coding sequence, were considered hits. For each data set, a list of hits was compiled, and based on each list, a consensus sequence was generated. Eight promoters (24 bp) were selected for No. 1575 and No. 2049 each, and primers containing these were purchased from Metabion. DNA templates for transcription were generated by PCR using primers containing the putative promoter sequences. After digestion with DpnI (ThermoFisher) DNA was purified. Quality was determined via agarose gel electrophoresis.

### 2.4. In Vitro Transcription Assay (IVT), Purification, and Analysis of RNA

Reactions with T7 or newly identified RNA polymerases were performed according to the HighYield T7 RNA Synthesis Kit from Jena Bioscience. After mixing, reactions were incubated at 20–50 °C for up to 240 min. Respective samples were pooled, and 2 U of Turbo™DNAse (ThermoFisher) were added, followed by an incubation at 37 °C for 30 min. The DNase treatment was stopped by addition of EDTA (VWR) to a final concentration of 25 mM. RNA was purified via Monarch RNA Cleanup Kit (NEB), and concentration was subsequently determined by UV/Vis (A260). A total of 200 ng RNA was mixed with 2X RNA loading dye (ThermoFisher) and heated at 95 °C for 2 min, followed by 1–3% agarose gel electrophoresis (SYBR Green I (ThermoFisher) stained, 7.8 V/cm, 60 min, 1X TBE buffer (ThermoFisher).

### 2.5. Fluorescent IVT Assay

In vitro transcriptions comparing T7 and newly characterized RNA polymerases with various promoters in a fluorescent setup were essentially performed as described above. In these IVT assays, the resulting mRNA product was the RNA aptamer F30-2xdBroccoli [46]. Additionally, DFHBI-1T (Sigma Aldrich) was added to a final concentration of 10 µM, and the reaction was brought up to 35 µL using nuclease-free water (Nalgene, Rochester, NY, USA). After mixing, the reactions were transferred into pre-cooled, clear-bottom, black 96-well plates, which were incubated at 37 °C using a Varioskan Lux 3020 microplate reader (ThermoFisher). Fluorescence was detected every 5 min (Ex/Em = 482 nm/505 nm) for 240 min using the Gel Doc XR+ documentation system (BioRad). A weak promoter–RNA polymerase interaction results in a lower fluorescence signal, while stronger interactions lead to higher signals.

### 2.6. dsRNA Content (Split Luciferase Complementation Assay via NanoBiT Technology)

To determine the dsRNA amount, mRNA samples were processed according to the Lumit dsRNA Detection Assay (Promega, Madison, WI, USA). The resulting dsRNA concentration (ng/mL) was used to calculate the dsRNA content (%) of each sample.

### 2.7. mRNA Integrity (Capillary Electrophoresis)

The purity and integrity of an mRNA was assessed using capillary gel electrophoresis with laser induced fluorescence (CGE-LIF) on a Sciex PA800 Plus device (SCIEX). Samples were diluted in formamide to a final concentration of 1–5 ng/µL and subsequently heated for 5 min. Prior each sample run, a bare fused silica capillary with a total length of 30.2 cm and an inner diameter of 50 μm was filled with a gel containing a polymer, 4.6 M urea, and the fluorescent dye SYBR Green II. The percentages of intact and degraded mRNA molecules were determined by calculation of the respective relative peak areas. A commercially available RNA ladder was used for size estimation of the mRNA samples.

## 3. Results

### 3.1. Bioinformatic Search of Genomic Meta-Database Provides a Large Set of Potential Enzymes

**Figure 1:**
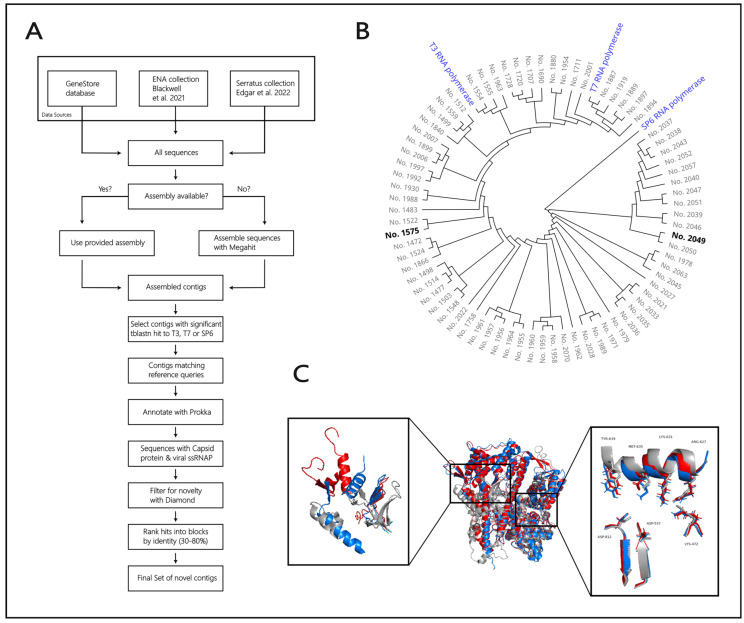
Bioinformatical design and creation of an RNA polymerase (RNAP) variant library and structural analysis of two variants. (**A**) Flow chart of RNAP sequence origin via genome mining. A final set of potential RNAP sequences (74) was generated by assembly, annotation and filtering of all genomic sequences (~2700) derived from three data sources [38,39]. (**B**) Circular phylogenetic cladogram of 77 genomic sequences. Three commercially available RNAPs (T3, T7, and SP6) are highlighted in blue. The two RNAP variants (No. 1575 and No. 2049) that were selected for further investigation are marked in bold black. (**C**) Structural comparison of RNAP No. 1575 (red) and RNAP No. 2049 (blue) to T7 RNAP (grey, PDB: 2PI5). The structure models of RNAP No. 1575 and No. 2049 were generated by AlphaFold [43,44] and the structures are superimposed on T7 RNAP. The right zoom box shows the active site of T7 RNAP, including seven important amino acid residues (Lys-472, Asp-537, Arg-627, Lys-631, Met-635, Tyr-639, and Asp-812). In the left zoom box, the promoter binding domains of T7 RNAP (AT-rich recognition loop, specificity loop, and intercalating β-hairpin loop) are represented.

Sequences from the genomic databases in the GeneStore of Proteineer GmbH were screened to identify not-characterized single-subunit RNA polymerases. The GeneStore database contains petabytes of sequences, including a broad spectrum of genomic databases, such as the European Nucleotide Archive [36] and the Joint Genome Institute Genome Portal [37], as well as the ENA Blackwell collection [38] and the Serratus collection [39] (Figure 1A).

The search entry for the analysis is the consensus sequence of 41 described RNA polymerases annotated for DNA-dependent RNA polymerase activity and mostly phage derived. The chosen sequences share only low sequence identity, which is intentional, as it increases the chance of identifying new polymerases. Especially, the sequence identity of the promoter-interacting N-terminal domain and the specificity loop is meant to vary greatly as these regions play a major role in dsRNA formation. All the selected sequences comprise the seven catalytic amino acids essential in the T7 RNA polymerase (Figure 1C).

After searching the GeneStore, the output sequences were assembled into contigs (contiguous sequences) using the MEGAHIT de novo assembly tool [40] (Figure 1B). Only contigs that provided a significant hit via the TBLASTN search algorithm with a default e-value cutoff to either the T7, SP6, or T3 phage single-subunit DNA-dependent RNA polymerase were further processed, resulting in approximately 2700 hits. Contigs considered a hit were annotated with Prokka [41]. Next, filtering for hit novelty, contigs containing annotations for a capsid protein and a single-subunit RNA polymerase (ssRNAP) of viral origin were used. These parameters were vital for ensuring the existence of a phage RNA polymerase and providing a potential promoter region upstream of the structural capsid protein, as the capsid genes belong to the late genes of the viral genome and, therefore, most likely are under the control of the promoter of the phage RNA polymerase [47]. Filtering was done with DIAMOND [42] and novelty was equivalent to the absence of any protein sequence from a commonly available source with 100% sequence identity. An amino acid length greater than 600 was a requirement to make sure that a full-length protein was detected. The filtered contigs were ranked by sequence identity to the T7 RNA polymerase (30–80%) in five blocks, and the final 74 sequences and their potential promoter regions (100 bp) were compiled from the top hits of each block. Protein structure modeling of sequences No. 1575 and No. 2049 via AlphaFold [43,44] and superimposition of the models on the structure of T7 RNA polymerase confirm a low structural similarity of the N-terminal domains and a high structural similarity of the catalytic residues (Figure 1C).

### 3.2. Expression Analysis of Potential RNA Polymerases Produced by E. coli

**Figure 2:**
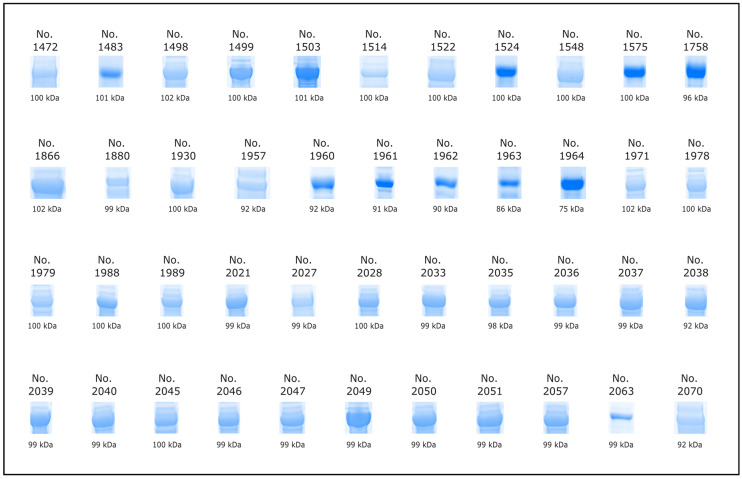
Expression analysis of 44 RNA polymerase (RNAP) variants. All RNAP variants were expressed in *E. coli* BL21(DE3), and crude lysate samples were analyzed via SDS-PAGE. An empty vector control (IPTG-induced cells harboring an empty pET28a vector) was used for the gels to determine host-derived background protein bands. The gel images show sections of gels at sizes relevant for the respective protein. The samples were collected after four hours of induction with 0.5 mM IPTG. The size of all proteins is given in kilodaltons (kDa) and indicated below each SDS gel.

A sequence analysis of the final 74 filtered candidate sequences was performed. The analysis revealed 10 homologous sequence pairs sharing a similarity of more than 98%. Therefore, only one of each pair was picked for further experiments. Eleven candidates seemed to have a deleterious effect on *E. coli,* and therefore, it was not possible to clone them into a vector. Out of the remaining 53 candidates, 44 putative RNA polymerases were produced by *E. coli* (Figure 2). Following optimization of the buffer system, 12 soluble proteins were obtained. These proteins were purified via gravity flow affinity chromatography (Ni-NTA, Qiagen) and were applied in further trials. For reactions determining the dsRNA content and mRNA integrity, proteins No. 1575 and 2049 were further purified by fast protein liquid chromatography (FPLC) (Supplementary Figure S1A,B).

### 3.3. Investigation of the Interaction Between Proposed Promoter Sequence and Potential RNA Polymerases via In Vitro Transcription Assays

For each of the 74 hit sequences, a potential promoter region of 100 bp was identified. These regions are upstream of the viral capsid protein detected in each genomic data set. For an RNA polymerase, interaction with its promoter region is essential; therefore, the first trials focused on the identification of the enzyme-specific corresponding promoter region. Template DNA comprising the various proposed promoter regions was generated. One of the 12 expressed and soluble proteins (No. 2049) produced full-length GFP RNA, coding for the green fluorescent protein, with various shorter transcripts, indicating activity but highly inefficient (Supplementary Figure S2). No. 2037, 2038, and 2051 generated very low amounts of RNA with a high number of abortive RNA species, while the remaining eight proteins displayed no enzymatic activity. Further trials were performed using the T7, T3, and SP6 promoter as the sequence homology implies possible activity on those sequences. In combination with the SP6 promoter, No. 2049 was able to produce the desired RNA with almost no by-products. In contrast, when combined with the T3 promoter, it failed to synthesize any RNA. These findings highlight the importance of identifying the appropriate promoter region and further research focused on optimizing the sequences to develop a strong promoter.

### 3.4. Sequence Optimization Based on Phage RNA Polymerase Core Region Contributes to Promoter Identification of RNA

**Figure 3:**
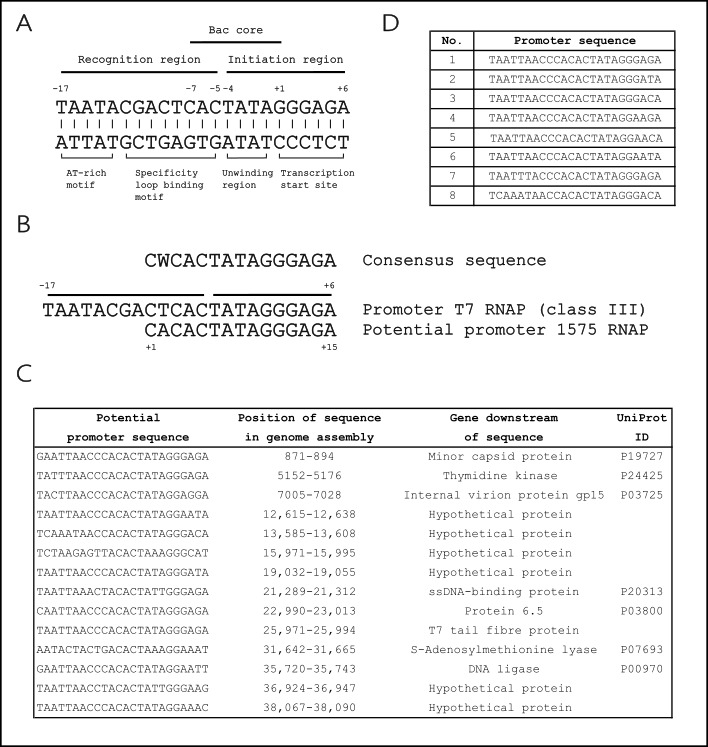
Expression analysis of 44 RNA polymerase (RNAP) variants. Identification of promoter sequences for RNA polymerase (RNAP) variant No. 1575. (**A**) Schematic representation of the class ΙΙΙ promoter for T7 RNA polymerase (position −17 to +6) (adapted from [8,10]). (**B**) Sequence alignment of the class ΙΙΙ promoter for T7 RNA polymerase and the potential promoter region of 100 bp for RNAP variant No. 1575 (+1 to +15). (**C**) List of 14 potential promoter sequences for RNAP variant No. 1575 and their genetic context. (**D**) List of final potential promoter sequences for RNAP variant No. 1575 selected for further investigation. The same lists were generated for RNAP variant No. 2049 (see Supplementary Figure S3A,B).

The T7 promoter (23 bp) consists of two major regions, the recognition and the initiation region (Figure 3A). The first one extends from −17 to −5 and includes the AT-rich motif (ATM) and the specificity loop binding motif (SLBM). Both motifs contribute to the reaction by interacting with the AT-rich recognition loop and the specificity loop of the RNA polymerase, thereby enabling the identification and binding of the polymerase to the promoter. The initiation region ranges from −4 to +6 and comprises the unwinding region, which is heavily involved in promoter melting, and the transcription start site (TSS). The positions +1, −1, −3, and −4 are conserved among the phage RNA polymerase promoters [8,10]. Moreover, the three promoters of T7, T3, and SP6 polymerases share a very similar core region (CACTATAG) reaching from −7 to +1 (bac core), implying a major function in bacteriophage-driven transcription [10,45].

Comparison of the T7 promoter core region to the proposed 100 bp promoter sequences of the soluble produced proteins No. 1575, 1758, 1957, 1960, 1961, 2037, 2038, 2039, 2040, 2045, 2049, and 2051 demonstrates varying alignment results. Regions for proteins No. 1758, 1957, 1960, and 1961 share low sequence identity (43.4 to 63.6%), with the T7 promoter and lack the bac core region. The putative promoter regions of No. 1575, 2037, 2038, 2039, 2040, 2045, 2049, and 2051 contain the entire bac core, with the promoter for No. 1575 having the highest similarity of 93.3%. Since RNA polymerase No. 2049 transcribes the full-length RNA under the control of the SP6 promoter and as No. 2037, 2038, 2039, 2040, 2045, and 2051 are genetically close to No. 2049, their promoters are compared to the SP6 promoter, and analysis reveals a sequence identity of 82.6%.

To find promoter sequences, the specificity loop binding motif represents an essential part. In the data set of No. 1575, the bac core and the transcription start site are present in the alignment, while the motifs interacting with the polymerase are absent (Figure 3B). These domains are outside of the proposed promoter region; hence, a more detailed search based on the bac core region in each genome assembly, representing the viral genomes, was necessary to identify the entire promoter sequence.

The new search was extended to 150 bp upstream of a gene and included specifically the positions −7 to −3 of the bac core region (CACTA) that are common in bacteriophage promoter consensus sequences [48]. This ensured that hits from this search are in areas of potential promoters. For protein No. 1575, for example, 14 hits were identified (Figure 3C). Alignment of all hits yields a consensus sequence termed promoter 1. Promoters 2 to 8 are generated by introducing substitutions naturally occurring in the corresponding genome assembly (Figure 3D). The same process was performed for the seven proteins No. 2037, 2038, 2039, 2040, 2045, 2049, and 2051 to compile a set of 8 promoter regions, as all associated proposed promoter sequences are very similar. The first promoter resembles the SP6 promoter. Promoters 2 and 3 are the consensus sequences, while promoters 4 to 8 display either one, two, or three substitutions (Supplementary Figure S3A,B). The search for proteins No. 1758, 1957, 1960, and 1961 yielded no hits. Even with alternative search entries no bacteriophage promoter-like sequences were detected.

### 3.5. RNAP Polymerases No. 1575 and No. 2049 Exhibit Low Production of dsRNA By-Products During In Vitro Transcription

**Figure 4:**
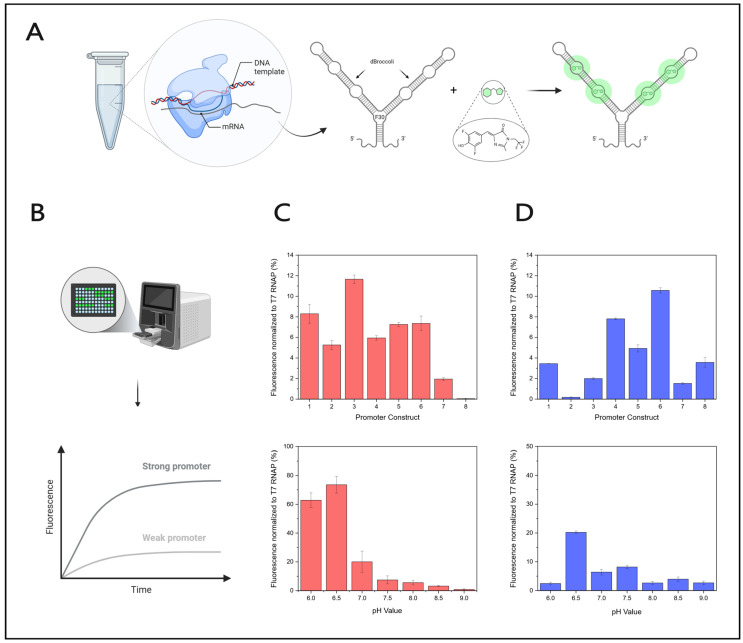
Fluorescent activity screening of RNA polymerases. (**A**) Schematic illustration of the production and visualization of the RNA aptamer F30-2xdBroccoli. The aptamer consists of two dimeric broccoli units (dBroccoli) and one stem region (F30) (adapted from [46]) and is produced via in vitro transcription (IVT). Upon binding of the dye DFHBI-1T (indicated in green) to the secondary structure of the RNA aptamer, the complex starts to fluoresce. This illustration was generated using BioRender.com. (**B**) IVTs, including DFHBI-1T, were carried out in a 96-well plate format, and the resulting fluorescence was measured over time via a multi-mode microplate reader. The fluorescence level indicates the production level of the RNA aptamer and, therefore, directly correlates with the strength of promoter binding and RNA polymerase (RNAP) activity. This image was created using BioRender.com. (**C**) RNAP No. 1575 activity normalized to T7 RNAP using different promoters 1–8 (top) and varying pH 6–9 with promoter 3 (below). (**D**) RNAP No. 2049 activity normalized to T7 RNAP using different promoters 1–8 (top) and varying pH 6–9 with promoter 6 (below).

The optimized promoter sequences were tested for the two proteins No. 1575 and 2049 (Table A1) using a fluorescent RNA aptamer screening setup. Among the proteins No. 2037, 2038, 2039, 2040, 2045, 2049, and 2051, RNA polymerase No. 2049 was selected as a representative for this group based on genetic similarity. Upon synthesis, the RNA aptamer forms a secondary structure consisting of two broccoli units and one stem region and starts to fluoresce once bound to DFHB-T1 (Figure 4A,B). In vitro transcriptions with RNA polymerase No. 1575 and the specific eight newly identified promoters show normalized fluorescence values ranging from 0.04 to 11.6% (normalized to T7 RNA polymerase). Promoter 3 (consensus with G->C substitution in position +5) has the highest value (11.6%) compared to all variants, including the consensus sequence (promoter 1) (8.3%). Promoter variants 2 (consensus with G->T substitution in position +5) and 4 (consensus with G->A substitution in position +3) exhibit lower values (5.3% and 5.9%) compared to promoter 1. Promoter versions with two substitutions at position +3 and +5 (promoters 5 and 6) are slightly less fluorescent in comparison to the consensus (promoter 1) (7.2 and 7.3%). The substitution in the promoter 7 (A->T substitution in position −13) decreases the fluorescence value to 2%. Promoter 8, being substituted in six positions (−17, −15, −14, −13, −11, and +5), demonstrates the lowest fluorescence (0.04%) (Figure 4C and Supplementary Figure S4A).

When analyzing polymerase No. 2049 using the SP6 promoter (promoter 1), a higher fluorescence (3.5%) is observed compared to the consensus sequence of protein No. 2045 (promoter 2) (0.18%). A slightly lower signal is obtained using the consensus sequence of proteins 2037, 2038, 2039, 2040, 2049, and 2051 (promoter 3), where about 2% are achieved. Substitutions within promoter 3, introducing changes at position +5 (promoter 4) or +2 (promoter 5), increase the values (7.8 and 4.9%, respectively). A double substitution in position +2 and +5 (promoter 6) shows the highest fluorescence levels (10.6%) among all tested promoters for RNA polymerase 2049. Similar substitutions in position +2 and +3 (promoter 7) do not have a beneficial effect and reduce fluorescence (1.5%). In vitro transcriptions using promoter 8, which contains three substitutions in positions +2, +3, and +5, display almost the same fluorescence as in vitro transcriptions employing the consensus sequence promoter (promoter 1) (3.6%) (Figure 4D and Supplementary Figure S4C). Subsequent experiments were carried out with promoter 3 for No. 1575 (1575-3) and promoter 6 for No. 2049 (2049-6).

Furthermore, potential cross-recognition of promoter sequences by the RNA polymerases applied in these experiments was investigated. Given that the sequence similarity between the T7 and the 2049-6 promoter within the specificity loop binding motif (SLBM), essential for efficient promoter, polymerase interaction, is 37.5%, and considering previous reports demonstrating high promoter specificity of RNA polymerases, where a single nucleotide change can result in a loss of transcriptional activity [49,50], T7 RNA polymerase and No. 2049 were not tested on each other’s promoters. Sequence similarity between the T7 and the 1575-3 promoter within the SLBM is 50%, with an overall sequence identity of 75%, suggesting a higher probability of cross-recognition compared to promoter 2049-6. Therefore, in vitro transcriptions were performed using both RNA polymerases and the corresponding promoters in all four combinations, employing DNA templates encoding 2xdBroccoli RNA (240 nt). Gel analysis reveals that neither RNA polymerase was able to produce the RNA aptamer when provided with the non-cognate promoter (Supplementary Figure S4E). Additionally, the stability of the RNA polymerases at different storage temperatures was investigated. Therefore, enzymatic activity was evaluated under identical in vitro transcription conditions after the enzymes had been stored for 27 days at 4 or −20 °C (Supplementary Figure S4F,G). Storage of No. 1575 at −20 °C, in contrast to at 4 °C, appears to impair catalytic activity, whereas No. 2049 retains similar activity, independent of the storage temperature. Therefore, RNA polymerase No. 1575, stored at 4 °C, and No. 2049, stored at −20 °C, were used for further experiments.

Next, the optimal pH value for the in vitro transcription using the two RNA polymerases No. 1575 and No. 2049 was determined. Reactions incubated at 37 °C for 4 h with polymerase No. 1575 and promoter 1575-3 at pH 6 display a high level of 62.8% normalized fluorescence. At pH 6.5, the value even rises to 73.6%. At higher pH values (7–9), the values drop (20–0.9%). The same experimental setup using polymerase No. 2049 and promoter 2049-6 shows that the fluorescence of the reactions at pH 6, 8, and 9 is in a similar low range (2.5–2.7%), while reactions at pH 7, 7.5, and 8.5 display higher values (4–8.2%). The highest value is observed when the pH value is set to 6.5 (20.2%) (Figure 4C,D; Supplementary Figure S4B,D). Further reactions were carried out at pH 6.5. To evaluate the processivity and the general production capabilities of the RNA polymerases, template DNA of different lengths was investigated. The DNA used for this study contained either the RNA aptamer 2xdBroccoli (240 nt), the genes for the green fluorescent protein (GFP; 764 nt), the firefly luciferase (FLuc; 1721 nt), or the CRISPR-associated protein 9 (Cas9; 4135 nt). The selected DNA templates are representatives of the typical size range of therapeutic mRNA products, especially the Cas9 mRNA is of interest as it is of similar size to the mRNAs of the COVID-19 19 vaccines. Accordingly, these templates were chosen to determine the mRNA integrity and dsRNA content and to determine how broadly the characteristics of the RNA polymerases tested can be applied. The in vitro transcription reactions were incubated at different temperatures (20–50 °C) for 3 hours each at their predetermined optimal pH value and their corresponding promoter. A temperature range of 37 to 45 °C proves to be the most effective for transcription with the newly identified RNA polymerases.

Regarding the yield, RNA polymerase No. 1575 is more efficient when supplied with shorter templates compared to longer ones (Table 2). When given the shortest DNA, the enzyme shows a yield of 7.3 mg/mL, which is higher than the standard T7 polymerase (4.1 mg/mL). For longer templates, the yield of No. 1575 decreases (1–5.9 mg/mL) and is lower compared to the standard enzyme (4.1–12.7 mg/mL). In combination with the Cas9 template, the yield increases slightly. The same effect can be detected for RNA polymerase 2049, although overall this enzyme displays a much lower yield (0.24–1.6 mg/mL). Its yield is maximized when the small 2xdBroccoli template is used (1.6 mg/mL) and declines with increasing template length. The integrity, measured via capillary electrophoresis, of the mRNA products generated by No. 1575 decreases (from 60.8 to 7.4%) as the length of the DNA template increases (240–4135 bp). In comparison to the mRNA integrity generated by the control T7 enzyme (from 98.5 to 57.2%), the integrity produced by No. 1575 is substantially lower. RNA polymerase No. 2049 transcribes mRNA with lower integrity than the control (49.5–95.1%) as well, except when the GFP template is used. In this case, the mRNA product synthesized by No. 2049 demonstrates a higher integrity (95.1%) compared to the control (91.2%). The acceptance of modified nucleotides by the not-characterized RNA polymerases was tested in our in vitro transcription setting in the following step. No. 1575 incorporates the clinically relevant Pseudo-UTP (Ψ), N1-methylpseudo-UTP (1mΨ), 5-methoxy-UTP (5moU), and 5-methyl-CTP (m5C), but with low yields. Due to the low incorporation, further analysis could not be carried out. Also, the analysis of the incorporation of modified nucleotides of enzyme No. 2049 was not performed. As the yields of this enzyme with unmodified nucleotides were very low, expectedly low yields were seen with modified nucleotides.

Furthermore, a key RNA polymerase feature, dsRNA by-product formation, was investigated. Surprisingly, the polymerase No. 1575 is able to decrease the dsRNA contamination to very low levels (0.001–0.007%) relative to the standard (0.12–0.51%). This would correspond to a 160-fold reduction compared to the T7 RNA polymerase for long RNAs (Cas9). For shorter RNAs (GFP, FLuc) the extent of the reduction is smaller (73–120-fold). The RNA polymerase No. 2049 also produces mRNA with much lower dsRNA levels (0.02–0.05%) in comparison to the standard. This enzyme achieves a 4-fold reduction for long RNA and 6–10-fold for shorter RNAs (Table 2; Supplementary Figure S5A–E).

## 4. Discussion

*Sequences obtained from genome mining*. Sequence analysis of the final 74 hits reveals that 56 share an identity with T7 RNA polymerase, between 30% and 80%. The remaining 18 hits have an identity percentage below or above this filter. Since these 18 hits should not be considered, parameters must be adjusted. Besides T7, there are other commercially available polymerases, such as SP6 RNA polymerase. Alignments of the 56 hits with SP6, however, demonstrated significant homology between additional 10 hits and this enzyme (>84%). As the SP6 polymerase is a well-studied enzyme [17,18], the filters need to be extended to ensure capturing hits within 30–80% identity to this polymerase as well. Moreover, 20 out of 74 hits show a high pairwise sequence similarity. To ensure that hits are not redundant, the filters must be redefined.

*Expression analysis of genomic sequences*. The analysis of the newly identified sequences presents various expression patterns. Most hits yielded proteins under the control of the T7 promoter using IPTG induction. Some hits showed expression via the PBAD promoter and induction by L-arabinose [51], while others could not be expressed. Reasons for this could be a metabolic burden induced by heterologous genes or defense mechanisms of the host. The overexpression of genes in a cell can lead to depletion of resources and appropriation of the protein biosynthesis machinery, potentially resulting in the cell’s death [52,53]. Furthermore, bacteria have mechanisms to resist viral infections, such as the CRISPR-Cas systems [54,55]. Since the hits are mostly phage derived, it could be possible that the cells eliminated the genes as they were recognized as viral DNA. Moreover, it must be stated that the throughput of genomic sequences and expressed proteins was low. Limiting the hits to 74 was crucial to enable feasibility in a small-scale lab setting. Yet this restriction, while necessary, could lead to the exclusion of not-yet-characterized enzymes. Additionally, many proteins were insoluble and not examined for activity, thereby further reducing the number of potential enzymes. As this study was neither designed nor executed as a high-throughput screening, the evaluation of expression efficiency in *E. coli* and the systematic optimization of expression strategies remained limited in scope. These candidates, No. 1575 and No. 2049, were prioritized because they (i) were obtained as soluble preparations, (ii) showed robust transcription activity in the initial IVT screens, (iii) exhibited the most favorable dsRNA-to-yield profile among the soluble candidates under our standardized assay conditions, and (iv) had identifiable promoter elements enabling further characterization. Moreover, expression of recombinant proteins can represent a toxic burden on the host cell, which further constrains the extent to which the production of these proteins can be enhanced [53]. It should be noted, however, that the remaining ten soluble proteins retain considerable potential as promising RNA polymerase candidates. To evaluate more sequences from the database, a system without limitations due to protein expression and purification is needed.

*Transcriptions with the proposed promoters.* The regions 100 bp upstream of a viral capsid gene potentially carry the corresponding promoters for each polymerase. Here the regions showed little to no activity in reactions with the specific enzyme, except for No. 2049. Capsids are structural proteins expressed late in the cycle, which are transcribed by the viral RNA polymerase [47]. Therefore, the promoter of the viral polymerase is upstream of the capsid gene. Here, in 11 out of 12 proteins, it is possible that the promoter was not included in the 100 bp. This could be a result of the promoters for late genes showing a variation of strength and position [56]. Some viral promoters are positioned up to 300 bp upstream, underlining the statement above. Some proteins produced only a little RNA with their regions, which could be the case due to the promoters initiating weak to medium transcription. Regarding the increased abortive transcription of No. 2049 with its region compared with the SP6 promoter, it must be stated that the region contains an additional 50 bp before the location of the ribosome binding site compared to SP6. These 50 bp may include regulatory elements, which could interfere with the full-length mRNA production. Here, activity for some proteins was demonstrated. Nevertheless, an improvement of the promoter identification process is required.

*Promoter screening with newly identified promoters*. As only one of the 100 bp upstream regions showed activity, a detailed analysis based on the bac core was performed and yielded different sets of newly identified promoters. The bac core is located in position −7 to +1 of the T7 promoter. This region is highly similar among the promoters of T7, T3, SP6, and K11, suggesting importance for phage promoter function. It was, therefore, chosen as the search entry.

The resulting hits for the RNA polymerases No. 1575 and 2049 were used to create a consensus, which was tested as a promoter. A mutation in the AT-rich motif (ATM) at position −13 of the consensus seems to weaken the interaction of promoter No. 1575. The ATM (−17 to −13) is of little importance for initiation but required for promoter recognition by T7 RNA polymerase [10]. Therefore, the deviation from the consensus is expected to destabilize the initiation complex. Mutations in the transcription start site (TSS, +1 to +6) were also investigated. The mutation of A->G in +2 increases fluorescence. Substitutions with guanosine in +2 appear to be beneficial for transcription with No. 2049, as is documented for T7 [8]. The next position examined was +3, showing reduced values. The first two positions of the TSS are sensitive to mutations, and the T7 shows a strong preference to start initiation with GTP [8,10]. While this preference is yet unclear, it has been suggested that stacking of GTPs may favor the incorporation of purines. Therefore, introducing a mutation in +3 may lead to a reduced initiation efficiency of No. 1575. Single mutations in +5 exhibit different results. Mutation of A->G displays an increased activity of the enzyme of No. 2049, as guanosine mutations proved to be advantageous [8]. An exchange of guanosine with thymine in +5 reduces the promoter interaction of No. 1575; meanwhile, substitution with cytosine increased promoter strength. It has been reported that mutations of guanosine in +5 of the T7 promoter with thymine or cytosine alter the promoter strength equally marginally [48]. These data suggest that, despite showing T7-like features, RNA polymerase No. 1575 prefers the mutation of G->C over G->T. When comparing the structures of cytosine and thymine, it becomes evident that thymine, unlike guanine, can form only two hydrogen bonds, while cytosine can form three, just like guanine. A stronger interaction may stabilize the promoter melting bubble. This is supported by the observation that many guanosines are located in the TSS [10]. Double nucleotide changes at the TSS in positions +2 and +3 or +3 and +5 also show decreased enzymatic activity, indicating weak promoter sequences. As a previous mutation in +3 decreased strength, it was anticipated that these sequences would be weak promoters. A double A->G mutation +2 and +5, however, resulted in a higher value compared to the consensus of No. 2049, indicating a preference for guanosine in the TSS [10]. A triple mutation in +2, +3, and +5 led to the same value as the consensus, carrying the beneficial mutations in +2 and +5 and the deleterious one in +3.

For both RNA polymerases (No. 1575 and 2049), a negative control promoter was generated. Promoter variant 8 of No. 1575 contains four mutations in the ATM, one in the specificity loop binding motif (SLBM) and one in the TSS. As the mutation in the ATM led to less fluorescence, it was expected that this promoter variant, in combination with No. 1575, would lead to a weak fluorescent signal. The SLBM of the T7 promoter (−12 to −5) is essential for RNA polymerase recognition [8]. It was anticipated that mutations in this motif would generate a weak promoter. For No. 2049 the consensus of the data set of No. 2045 was selected as the negative control (promoter 2), enabling minimal fluorescence with No. 2049. This was expected as five out of eight nucleotides of the SLBM were exchanged, and therefore, specificity for No. 2049 was lost.

*pH and temperature.* The observation that No. 1575 and No. 2049 are most active at pH 6.5 suggests that they originate from a slightly more acidic environment, such as springs or skin [57,58]. Using these enzymes at a lower pH in reactions can be beneficial, as RNA displays a higher stability at lower pH values [59]. Regarding the temperature, both enzymes achieve the highest yield at 37–45 °C, implying a mesophilic origin. Using BLAST+ 2.17.02, both sequences were compared to the ones stored in the database. No. 1575 shares a 99.96% identity with the genome of Vibrio phage JSF35. This is plausible as it is derived from a source named “Within-host evolution of Vibrio cholerae using metagenomics”. For No. 2049, an identity similarity of 91.76% to the genome of Escherichia phage NTNC80A was detected. Escherichia bacteria, as well as Vibrio cholerae, are adapted to a mesophilic environment, which is in line with our findings [60,61].

*mRNA yield and integrity.* RNA polymerase No. 1575 yielded a higher amount of short RNA (Broccoli Aptamer) relative to the T7 RNA polymerase, suggesting higher processivity than T7. Processivity refers to the number of catalytic reactions an enzyme can perform before releasing its substrate. Here, processivity is defined by the number of nucleotides incorporated by the RNA polymerase during a single template-binding event. Hence, high RNA yield and integrity are indicative of high processivity. The integrity of RNA produced by No. 1575 from 2xdBroccoli DNA template was assessed by agarose gel electrophoresis (Supplementary Figure S4E). This analysis was performed because the RNA aptamer is small in size (240 nt), making its integrity less critical and allowing only an approximate evaluation. In contrast, the integrity of the larger RNAs is more relevant, as their lengths more closely resemble those of mRNA therapeutics. Analysis of the gel demonstrates that No. 1575 synthesized the RNA aptamer with minimal by-product formation, especially at pH 6.5. When longer DNA templates are used (GFP, FLuc, Cas9), however, the yield produced by No. 1575 decreases, indicating a decreased processivity compared to T7. Having a lower processivity, the enzyme would detach from the DNA more often than T7, generating more abortive transcripts. The low mRNA integrity produced by No. 1575 is consistent with the reduced processivity on longer DNA templates. This suggests that No. 1575 may possess higher activity on short DNA templates, likely due to the limited ability to remain associated with longer DNA sequences. No. 2049 generates lower yields and less RNA integrity compared to T7 RNA polymerase as well. However, the RNA integrity generated by No. 2049 is notably higher than that produced by No. 1575. These results could imply that No. 2049 transcribes more slowly and stays attached to the DNA longer than No. 1575. Both enzymes show a lower processivity on medium to long templates, while on short templates, No. 1575 is more processive compared to T7.

*Modified nucleotides.* No. 1575 can incorporate clinically relevant base-modified nucleotides (NTPs). Even though it can utilize modified NTPs, the processivity is very low, yielding only a little modified mRNA. Low RNA yields with modified NTPs, a common event, could be the result of No. 1575 recognizing modified NTPs less efficiently than unmodified [62,63]. In contrast to T7 or Sy5 mutants, No. 1575 may have difficulties recognizing 2’-ribose modifications, as it exhibits a tyrosine (Y647) at the structural position of Y639 in the T7 and a histidine (H792) at position H784 [64,65]. After improving the RNA yield and subsequent evaluation of its capacity to incorporate modified nucleotides efficiently, this polymerase may be applied in mRNA vaccine production, as these vaccines often depend on the incorporation of modified nucleotides. The extremely low RNA yield of No. 2049 severely limited the review of its ability to use modified NTPs, and further investigation of the use of modified NTPs is needed.

*dsRNA content.* The decrease in dsRNA content in all assays using No. 1575 compared to T7 RNA polymerase is significant. Depending on the DNA template, dsRNA levels were decreased by 73- to 160-fold, indicating a possible sequence-dependent effect. When given the DNA template for GFP, No. 1575 generated a 120-fold lower dsRNA content than the T7 RNA polymerase while producing approximately half the amount of RNA. This observation suggests improved performance on short DNA templates. For medium-sized RNAs, No. 1575 achieved a 73-fold reduction in dsRNA. The comparatively lower decrease in dsRNA observed with the FLuc DNA template is consistent with the reduced RNA yield, indicating a lower activity on that specific sequence. The highest dsRNA reduction (160-fold) was achieved by No. 1575 in combination with the Cas9 DNA template. However, it must be noted that barely any full-length Cas9 RNA was produced, despite a higher RNA yield compared to in vitro transcription reactions using DNA templates for GFP, FLuc, or the RNA aptamer. This implies a generation of abortive transcripts and that no RNA for dsRNA formation via 3′-extension was available. Overall, the dsRNA content was below 0.008%, highlighting the enzyme’s activity with negligible dsRNA generation. No. 2049 generated lower amounts of dsRNA compared to T7 RNA polymerase, although the reduction was less pronounced than that detected in reactions with No. 1575. On short, medium, and long DNA templates, No. 2049 enabled a four- to tenfold decrease in dsRNA. These findings suggest that, similar to No. 1575, this enzyme possesses characteristics that favor minimal dsRNA production. Although the RNA yield produced by No. 1575 and No. 2049 was substantially lower compared to the yield of T7 RNA polymerase on most DNA templates, the reduction in dsRNA content observed with No. 1575 and No. 2049 in direct comparison to T7 RNA polymerase highlights the potential of the newly identified RNA polymerases as early-stage candidates. Despite this advantage, these enzymes are not yet suitable for commercial use and will likely require engineering to be acceptable in an industrial setting. Nevertheless, the overall characteristics make them prime candidates for further optimization.

The main reasons for dsRNA formation are the mRNA 3′-extension or abortive cycling [22,23]. As the initiation complex (IC) is associated with abortive cycling, mutations stabilizing the elongation complex (EC) are assumed to minimize dsRNA. The amino acid glycine in position 47 in the T7 RNA polymerase is reported to be part of the structural rearrangement taking place during the transition from IC to EC and, thereby, to be involved in dsRNA generation. A double mutant carrying the mutation G47A, stabilizing the EC, and an insertion of glycine at position 884 reduces dsRNA to 0.007% [12]. Sequence alignment of the T7 RNA polymerase with No. 1575 and No. 2049 reveals glycine residues in the newly characterized polymerases that align to the glycine in position 47 described above. An imposition of the protein models (EC of T7, No. 1575 and No. 2049) shows all three glycines at the same location. The single mutations A70Q and K180E, the double mutation F162S/A247T, and the triple mutation A70Q/F162S/K180E in T7 RNA polymerase have likewise been identified to reduce dsRNA as well [66]. At all mutation sites, No. 1575 shares the same amino acid as T7 RNA polymerase, whereas No. 2049 retains only one of the four conserved amino acids of the T7 RNA polymerase (F162). The other three amino acids differ (A70N, K180H, A247S). Structural analysis of the protein models for No. 1575 and No. 2049, superimposed onto the structure of T7 RNA polymerase, determined via X-ray diffraction (PDB: 2PI5), reveals that the amino acids of No. 1575 and No. 2049 described above and identified in the sequence alignment are located at the same site and proximity as the mutation sites in the T7 RNA polymerase (A70, F162, K180, A247). These findings indicate that the reduction in dsRNA observed for the newly identified enzymes is not due to these positions. An examination via nuclear magnetic resonance (NMR) spectroscopy or X-ray crystallography of the IC formed by the newly identified enzymes and their promoters is needed to determine the underlying mechanisms.

The RNA polymerase of *Pseudomonas fluorescens* phage VSW-3 has been reported to produce decreased levels of dsRNA (0.001–0.0045%) relative to T7 RNA polymerase. These minimal levels of dsRNA are comparable to our findings. For the VSW-3 RNA polymerase, a missing RNA-dependent activity is likely the reason for the reduction [67]. An RNA transcript showing terminal secondary structure (sgRNA) was employed in this study to investigate the capacity of the two RNA polymerases (T7 and VSW-3) to facilitate 3′-end extension. Given that the T7 RNA polymerase is capable of self-templating on 3′-end hairpin RNA structures [27], the resulting sgRNA was expected to display 3′-end extensions upon sequence analysis, which was confirmed. In contrast, the VSW-3 RNA polymerase synthesized sgRNA lacking detectable 3′-end extensions. Moreover, the VSW-3 polymerase requires more time (12-fold) than the T7 to achieve an equivalent RNA yield, an effect that was not observed for the enzymes studied here. In contrast, No. 1575 is able to produce more RNA than T7 on short DNA templates. Experiments concerning the RNA-dependent activity of No. 1575 and No. 2049 should be conducted to investigate their capability to generate less dsRNA.

*Future perspectives*. DNA template characteristics, such as size and structure, can influence both the integrity of the mRNA and dsRNA formation. In the present study, however, only the promoter sequences were changed, and therefore, the overall architecture of the templates remains similar. A situation in which template length can play a more decisive role is demonstrated by larger DNA templates, which are generally associated with abortive transcription, potentially leading to increased dsRNA content and lower yield of full-length mRNA. We believe that an elaborate investigation of the effect of template DNA on the formation of dsRNA would be out of scope for an initial identification and characterization of RNA polymerases. This, however, will be required as a follow-up study, investigating the capability of the RNA polymerases to deal with different topological and sequence features of the template DNA.

Regarding the limited assessment of RNA polymerase storage stability, it should be emphasized that these experiments do not represent a comprehensive evaluation of enzyme stability. This is primarily because time-zero references were not conducted for every enzyme batch. Moreover, batch-to-batch variations in transcriptional activity further limit the reliability of quantitative comparisons. To enable the application of these RNA polymerases in manufacturing processes, a comprehensive study on enzyme stability (including defined storage conditions, time-zero baselines, multiple time points, and benchmarking against commercial T7 RNAP) will be essential.

Further improvement in overall protein purity will also be necessary. The purity levels obtained for the newly identified enzymes used in these experiments do not yet meet the standards demanded for application in therapeutic mRNA manufacturing. Future studies should therefore focus on more extensive enzyme purity assessment.

These newly characterized RNA polymerases extend the toolbox available for mRNA manufacturing due to their reduced dsRNA generation. However, as demonstrated in this study, their overall RNA yields do not reach those achieved by the T7 RNA polymerase, a critical parameter for efficient RNA production. The T7 RNA polymerase-based transcription system is widely regarded as a robust and attractive platform that is constantly improved. Nevertheless, it should be considered that many commercially available T7 RNA polymerases are not wild-type enzymes but engineered variants, improved for yield, efficiency, and incorporation of nucleotide analogs [20,35]. In contrast, the RNA polymerases identified in this study are directly derived from genomes and, therefore, constitute wild-type RNA polymerases, representing low dsRNA-generating alternatives and providing substantial potential for further enzyme engineering similar to that applied to the T7 RNA polymerase. Targeted engineering combined with process optimization may increase the yields of the newly characterized RNA polymerases, allowing a more direct performance comparison with T7 RNA polymerase. Future studies should compare these RNA polymerases using decision criteria, such as mRNA integrity, dsRNA content, full-length RNA yield, robustness across template length, and scalability. To conclude, wild-type RNA polymerases such as No. 1575 and No. 2049 generate substantially low dsRNA content, thereby offering new opportunities for the development of the next generation of RNA manufacturing.

## Figures and Tables

**Table 1:** Reagents, devices, and biological resources.

Name	Cat. No.	Manufacturer
Monarch RNA Cleanup Kit	T2050L	NEB Inc., Ipswich, MA, USA
GeneJET PCR Purification Kit	K0702	Thermo Scientific™, Waltham, MA, USA
GeneJET Plasmid Miniprep Kit	K0503	Thermo Scientific™
HighYield T7 RNA Synthesis Kit	RNT-101	Jena Bioscience GmbH, Jena, Germany
Lumit dsRNA Detection Assay	W2041	Promega, Madison, WI, USA
DpnI Restriction Enzyme	ER1701	Thermo Scientific™
TURBO™ DNase	AM2238	Thermo Scientific™
SYBR™ Green I	S7563	Thermo Scientific™
SYBR™ Green II	S7564	Thermo Scientific™
Century™-Plus RNA Marker	AM7145	Thermo Scientific™
DFHBI-1T	SML2697-5MG	Sigma-Aldrich
Varioskan Lux 3020 Microplate Reader	n/a	Thermo Scientific™
Sciex PA800 Plus Device	n/a	SCIEX, Framingham, MA, USA
BL21(DE3) Competent *E. coli*	C2527H	NEB Inc.
pET-29b(+)-RNA Polymerase Sequences	n/a	Twist Bioscience, South San Francisco, CA, USA

**Table 2:** Summary of various parameters investigated for the two RNA polymerases (RNAPs) No. 1575 and No. 2049 in direct comparison to RNAP T7. Apt = RNA Aptamer, GFP = GFP mRNA, FLuc = FLuc mRNA, Cas9 = Cas9 mRNA.

Parameter	RNAP T7				RNAP No. 1575				RNAP No. 2049			
Promoter	TAATACGACTCACTATAGGGAGA				TAATTAACCCACACTATAGGGACA				TATTTACTGGACACTATAGGAGGA			
pH Value	7.5				6.5				6.5			
Template	Apt	GFP	FLuc	Cas9	Apt	GFP	FLuc	Cas9	Apt	GFP	FLuc	Cas9
Yield (mg RNA/mL IVT)	4.1	12.7	10.9	7.4	7.3	5.9	1.0	3.0	1.6	0.4	0.31	0.24
Integrity (%)	n/a	91.2	98.5	57.2	n/a	60.8	18.1	7.4	n/a	95.1	51.7	49.5
dsRNA (%)	n/a	0.12	0.51	0.16	n/a	0.001	0.007	0.001	n/a	0.02	0.05	0.04
Modified Nucleotides	Pseudo-UTP, N1-methylpseudo-UTP, 5-methoxy-UTP, 5-methyl-CTP				Pseudo-UTP, N1-methylpseudo-UTP, 5-methoxy-UTP, 5-methyl-CTP				Not applicable due to insufficient yields			

## Data Availability

The raw data supporting the conclusions of this article will be made available by the authors on request.

## Supplementary Materials

The following supporting information can be downloaded at: https://www.mdpi.com/article/10.3390/microorganisms14030564/s1, Supplementary Figure S1A: 7.5% SDS gels of cultivation and purification samples of protein No. 1575. M = protein marker, OC = Overnight culture, MC = Main culture, 0, 4, 16 = time of cultivation, Son = Sonication, Sol = Soluble fraction, In = Insoluble fraction, HisTrap FF = Ni^2+^ sepharose column, HiTrap Q = Anion exchange column, FT = Flow-through fraction of the respective column, W = Wash fraction of the respective column, A4–C9 = Elutions of the of the respective column. The soluble fraction after sonication was applied to the HisTrap FF column. Elutions A4–A8 were used as samples for the HiTrap Q column. Elutions B2 and B3 were pooled and transferred into the storage buffer. Supplementary Figure S1B: 7.5% SDS gels of cultivation and purification samples of protein No. 2049. M = protein marker, OC = Overnight culture, MC = Main culture, 0, 4, 16 = time of cultivation, Son = Sonication, Sol = Soluble fraction, In = Insoluble fraction, HisTrap FF = Ni^2+^ sepharose column, HiTrap Q = Anion exchange column, FT = Flow-through fraction of the respective column, W = Wash fraction of the respective column, A5–C7 = Elutions of the of the respective column. The soluble fraction after sonication was applied to the HisTrap FF column. Elutions A6–A8 were used as samples for the HiTrap Q column. Elutions B8 and B9 were pooled and transferred into the storage buffer. Supplementary Figure S2: 2% agarose gel of purified mRNA samples of IVTs performed with T7 RNA polymerase, protein No. 2049 on a DNA template with the T7 or the 2049 promoter and the gene for GFP. The ladder is Century Plus marker. N = 1. Supplementary Figure S3A: List of 14 potential promoter sequences for RNAP variant No. 2049 and their genetic context. Supplementary Figure S3B: List of final potential promoter sequences for RNAP variant No. 2049 selected for further investigation. Supplementary Figure S4A: Activity of RNAP No. 1575 using different promoters 1–8 at pH 7.5 for 240 min measured in fluorescence (RFU). N = 3. Supplementary Figure S4B: Activity of RNAP No. 1575 using promoter 3 at various pH values for 240 min measured in fluorescence (RFU). N = 3. Supplementary Figure S4C: Activity of RNAP No. 2049 using different promoters 1–8 at pH 7.5 for 240 min measured in fluorescence (RFU). N = 3. Supplementary Figure S4D: Activity of RNAP No. 2049 using promoter 6 at various pH values for 240 min measured in fluorescence (RFU). N = 3. Supplementary Figure S4E: 10% TBE-Urea gel of purified mRNA samples (200 ng) of IVTs performed at pH 6.5 or 7.5 with T7 RNA polymerase or protein No. 1575 on a DNA template with the T7 or the 1575-3 promoter and the gene for 2xdBroccoli. The ladder is Century Plus marker. First, the Gel was stained with DFHBI-1T (40 µM) for detection of 2xdBroccoli and subsequently with SYBR Gold for detection of total RNA. N = 1. Supplementary Figure S4F: RNAP No. 1575 activity using promoter 3 at pH 7.5 for 240 min measured in fluorescence (RFU). The RNA polymerases applied were stored 27 days at the respective temperature before used in fluorescent in vitro transcription assays (IVTs). The right panel depicts the same data as the left panel, but without the data obtained for T7 RNAP to highlight the difference in RFU of IVTs with No. 1575 stored at different temperatures (−20 °C or 4 °C). N = 3 for T7 RNAP and No. 1575 stored at −20 °C, N = 1 for No. 1575 stored at 4 °C. Supplementary Figure S4G: RNAP No. 2049 activity using promoter 6 at pH 7.5 for 240 min measured in fluorescence (RFU). The RNA polymerases applied were stored 27 days at the respective temperature before used in fluorescent in vitro transcription assays (IVTs). The right panel depicts the same data as the left panel, but without the data obtained for T7 RNAP to highlight the difference in RFU of IVTs with No. 2049 stored at different temperatures (−20 °C or 4 °C). N = 3 for T7 RNAP and No. 2049 stored at −20 °C, N = 1 for No. 2049 stored at 4 °C. Supplementary Figure S5A: RNA yield of 2xdBroccoli mRNA produced by T7 RNAP (ctrl), RNAP No. 1575 and RNAP No. 2049 at 37 °C. N = 3. Supplementary Figure S5B: RNA yield, dsRNA content and mRNA integrity of GFP mRNA produced by T7 RNAP (ctrl), RNAP No. 1575 and RNAP No. 2049 at 37, 40 and 45 °C. N = 3. Supplementary Figure S5C: RNA yield, dsRNA content and mRNA integrity of FLuc mRNA produced by T7 RNAP (ctrl), RNAP No. 1575 and RNAP No. 2049 at 37, 40 and 45 °C. N = 3. Supplementary Figure S5D: RNA yield, dsRNA content and mRNA integrity of Cas9 mRNA produced by T7 RNAP (ctrl), RNAP No. 1575 and RNAP No. 2049 at 37, 40 and 45 °C. N = 3. Supplementary Figure S5E: 3% agarose gel of GFP mRNA produced by T7 RNAP (ctrl), RNAP No. 1575 with Pseudo-UTP, N1-methylpseudo-UTP, 5-methoxy-UTP, 5-methyl-CTP. N = 1.

## Abbreviations

The following abbreviations are used in this manuscript:

1mΨ	N1-methylpseudo-UTP
5moU	5-methoxy-UTP
AI	Artificial intelligence
ATM	AT-rich motif
BLAST	Basic local alignment tool
bp	Base pairs
Cas9	CRISPR-associated protein 9
CGE-LIF	Capillary gel electrophoresis with laser-induced fluorescence
CRISPR	Clustered regularly interspaced short palindromic repeats
DFHBI-1T	3,5-Difluoro-4-hydroxybenzylidene imidazolinone 1T
dsRNA	Double-stranded RNA
EC	Elongation complex
EDTA	Ethylenediaminetetraacetic acid
FLuc	Firefly luciferase
FPLC	Fast protein liquid chromatography
GFP	Green fluorescent protein
GTP	Guanosine triphosphate
IC	Initiation complex
IPTG	Isopropyl β-d-1-thiogalactopyranoside
IVT	In vitro transcription
LB	Lysogeny broth
m5C	5-methyl-CTP
nt	Nucleotides
NTD	N-terminal domain
NTPs	Nucleoside triphosphates
PAGE	Polyacrylamide gel electrophoresis
pDNA	Plasmid DNA
RNAP	RNA polymerase
SDS	Sodium dodecyl sulfate
SLBM	Specificity loop binding motif
ssRNAP	Single-subunit RNA polymerase
TBLASTN	Translated basic local alignment tool in nucleotide search
TSS	Transcription start site
Ψ	Pseudo-UTP

## Appendix A

**Table A1:** Amino acid sequences of the newly identified RNA polymerases No. 1575 and No. 2049.

**RNA Polymerase**	Amino Acid Sequence
No. 1575	ANVIKPESHNFSDISAAILPFNVLADSYGEALAAEQLMLEHESYQLGEARFIKAMERQVERGEVSDNAVAKPLLDTLIPALASRITEFVEMKQRGKPHVSKGYFAMIKPESAAFIIVKTTLNILAKEESVPVQRVAMAIGGNIEDEIRFGRIRDEEIKHFKERVKPNLDKRNGFIYKKAYMEAVEAGMQDKGELNSTHEAWEKDVKFHVGIRAIEMLIEATGMVQLERKFKGIPDKDHEALHLAPEYVEKLTNRAHALAGISPMYQPMIVKPKRWTGVQGGGYWAKGRRPLNLIRVGSKRALDRYRQVDMPEVYDAINTIQETAWRINKDVLAVVNNVVTWANCPVEDVPSIDKLALPEKPEDIDNNEESLKKWKKAAAAIYRKEKARQSRRISLEFALSQANKFSKYNEIYFPYNMDWRGRVYAIPMFNPQGNDMVKGLLTFAKKVPVGIDGGYWLAVHGANCAGVDKVSLEDRVKWVNDNEANIIASAEAPLDFTWWAEQDSPFCFLAFCFEWAAYVKAGKKPSFESSLPLAFDGTCSGLQHFSAMLRDEIGGAAVNLLPADKPQDIYDIVAVKVNEVLRDLVIRGTEDEMQTLEDKKTGEITERLVLGTRTLAAQWLEYGVTRSVTKRSVMTLAYGSKEYGFADQVFEDTVIPAIDNGKGAMFTEPSQACRFMAKLIWDAVSKTVVAAVEAMQWLQSAAKLVSSEVKDKKSGEILKHAMPVHWTTPNGFPVWSEYYKQEQKRIDCVILGTHRMALTINIRDKKEIDAAKQTSGIAPNFVHSMDASHLQMTVNKCFKVYGIHSFAMIHDSFGCHAGFASKMFRAVRETMVETYEEHDVIQEFYNQFEQQLHESQIEKMPVLPRKGNLELREILKSLYTFS
No. 2049	QDLHAIQLQLEEEMFNGGIRRFEADQQRQIASGNESDTAWNRRLLSELIAPMAEGIQAYKEEYEGKRGRAPRALAFINCVENEVAAYITMKIVMDMLNTDVTLQAIAMNVADRIEDQVRFSKLEGHAAKYFEKVKKSLKASKTKSYRHAHNVAVVAEKSVADRDADFSRWEAWPKDTLLQIGMTLLEILENSVFFNGQPVFLRTLRTNGGKHGVYCLQTSEHVGEWITAFKEHVAQLSPAYAPCVIPPRPWVSPFNGGFHTEKVASRIRLVKGNREHVRKLTKKQMPAVYKAVNALQATKWQVNKEVLQVVEDVIRLDLGYGVPSFKPLIDRENKPANPVPVEFQHLRGRELKEMLTPEQWQAFINWKGECTKLYTAETKRGSKSAATVRMVGQARKYSQFDAIYFVYALDSRSRVYAQSSTLSPQSNDLGKALLRFTEGQTINSTEALKWFLVNGANNWGWDKKTFDVRTANVLDGEFQDMCRDIAADPLTFTQWVNADSPYGFLAWCFEYARYLDALDEGTQDQFVTHLPVHQDGSCSGIQHYSAMLRDEVGAKAVNLKPSDSPQDIYGAVAQVVIQKNYAYMNADDAETFTSGSVTLTGAELRSMAGAWDMIGITRGLTKKPVMTLPYGSTRLTCRESVIDYIVDLEEKEAQKAIAEGRTANPVHPFDNDRKDYLTPGAAYNYMTALIWPSISEVVKAPIVAMKMIRQLARFAAKRNEGLEYTLPTGFILQQKIMATDMLRVSTCLMGEIKMGLQIETDVVDETAMMGAAAPNFVHGHDASHLILTVCDLVDKGITSIAVIHDSFGTHAGRTADLRDSLRAEMVKMYQGRNALQQLLDEHEERWLVDTGIQVPEQGDFDLNEILVSDYCFA

## References

[1] Al Fayez N., Nassar M.S., Alshehri A.A., Alnefaie M.K., Almughem F.A., Alshehri B.Y., Alawad A.O., Tawfik E.A. (2023). Recent Advancement in mRNA Vaccine Development and Applications. Pharmaceutics.

[2] Baden L.R., El Sahly H.M., Essink B., Kotloff K., Frey S., Novak R., Diemert D., Spector S.A., Rouphael N., Creech C.B. (2021). Efficacy and Safety of the mRNA-1273 SARS-CoV-2 Vaccine. N. Engl. J. Med..

[3] Polack F.P., Thomas S.J., Kitchin N., Absalon J., Gurtman A., Lockhart S., Perez J.L., Pérez Marc G., Moreira E.D., Zerbini C. (2020). Safety and Efficacy of the BNT162b2 mRNA Covid-19 Vaccine. N. Engl. J. Med..

[4] Sahin U., Karikó K., Türeci Ö. (2014). mRNA-based therapeutics--developing a new class of drugs. Nat. Rev. Drug Discov..

[5] Zhang C., Maruggi G., Shan H., Li J. (2019). Advances in mRNA Vaccines for Infectious Diseases. Front. Immunol..

[6] Jackson N.A.C., Kester K.E., Casimiro D., Gurunathan S., DeRosa F. (2020). The promise of mRNA vaccines: A biotech and industrial perspective. npj Vaccines.

[7] Borkotoky S., Murali A. (2018). The highly efficient T7 RNA polymerase: A wonder macromolecule in biological realm. Int. J. Biol. Macromol..

[8] Sousa R., Mukherjee S. (2003). T7 RNA polymerase. Prog. Nucleic Acid Res. Mol. Biol..

[9] Cheetham G.M., Steitz T.A. (2000). Insights into transcription: Structure and function of single-subunit DNA-dependent RNA polymerases. Curr. Opin. Struct. Biol..

[10] Padmanabhan R., Sarcar S.N., Miller D.L. (2020). Promoter Length Affects the Initiation of T7 RNA Polymerase In Vitro: New Insights into Promoter/Polymerase Co-evolution. J. Mol. Evol..

[11] Da L.-T., Chao E., Duan B., Zhang C., Zhou X., Yu J. (2015). A Jump-from-Cavity Pyrophosphate Ion Release Assisted by a Key Lysine Residue in T7 RNA Polymerase Transcription Elongation. PLoS Comput. Biol..

[12] Dousis A., Ravichandran K., Hobert E.M., Moore M.J., Rabideau A.E. (2023). An engineered T7 RNA polymerase that produces mRNA free of immunostimulatory byproducts. Nat. Biotechnol..

[13] Macdonald L.E., Zhou Y., McAllister W.T. (1993). Termination and slippage by bacteriophage T7 RNA polymerase. J. Mol. Biol..

[14] Calvopina-Chavez D.G., Gardner M.A., Griffitts J.S. (2021). Engineering Efficient Termination of Bacteriophage T7 RNA Polymerase Transcription. G3.

[15] McGraw N.J., Bailey J.N., Cleaves G.R., Dembinski D.R., Gocke C.R., Joliffe L.K., MacWright R.S., McAllister W.T. (1985). Sequence and analysis of the gene for bacteriophage T3 RNA polymerase. Nucleic Acids Res..

[16] Basu S., Sarkar P., Adhya S., Maitra U. (1984). Locations and nucleotide sequences of three major class III promoters for bacteriophage T3 RNA polymerase on T3 DNA. J. Biol. Chem..

[17] Krieg P.A., Melton D.A. (1987). In vitro RNA synthesis with SP6 RNA polymerase. Methods Enzymol..

[18] Stump W.T., Hall K.B. (1993). SP6 RNA polymerase efficiently synthesizes RNA from short double-stranded DNA templates. Nucleic Acids Res..

[19] Tabor S. (2001). Expression using the T7 RNA polymerase/promoter system. Curr. Protoc. Mol. Biol..

[20] Lyon S., Gopalan V. (2018). A T7 RNA Polymerase Mutant Enhances the Yield of 5′-Thienoguanosine-Initiated RNAs. Chembiochem.

[21] Milligan J.F., Groebe D.R., Witherell G.W., Uhlenbeck O.C. (1987). Oligoribonucleotide synthesis using T7 RNA polymerase and synthetic DNA templates. Nucleic Acids Res..

[22] Cazenave C., Uhlenbeck O.C. (1994). RNA template-directed RNA synthesis by T7 RNA polymerase. Proc. Natl. Acad. Sci. USA.

[23] Triana-Alonso F.J., Dabrowski M., Wadzack J., Nierhaus K.H. (1995). Self-coded 3′-extension of run-off transcripts produces aberrant products during in vitro transcription with T7 RNA polymerase. J. Biol. Chem..

[24] Arnaud-Barbe N., Cheynet-Sauvion V., Oriol G., Mandrand B., Mallet F. (1998). Transcription of RNA templates by T7 RNA polymerase. Nucleic Acids Res..

[25] Mu X., Greenwald E., Ahmad S., Hur S. (2018). An origin of the immunogenicity of in vitro transcribed RNA. Nucleic Acids Res..

[26] Lenk R., Kleindienst W., Szabó G.T., Baiersdörfer M., Boros G., Keller J.M., Mahiny A.J., Vlatkovic I. (2024). Understanding the impact of in vitro transcription byproducts and contaminants. Front. Mol. Biosci..

[27] Gholamalipour Y., Karunanayake Mudiyanselage A., Martin C.T. (2018). 3′ end additions by T7 RNA polymerase are RNA self-templated, distributive and diverse in character-RNA-Seq analyses. Nucleic Acids Res..

[28] Vlatkovic I. (2021). Non-Immunotherapy Application of LNP-mRNA: Maximizing Efficacy and Safety. Biomedicines.

[29] Heil F., Hemmi H., Hochrein H., Ampenberger F., Kirschning C., Akira S., Lipford G., Wagner H., Bauer S. (2004). Species-specific recognition of single-stranded RNA via toll-like receptor 7 and 8. Science.

[30] Karikó K., Muramatsu H., Welsh F.A., Ludwig J., Kato H., Akira S., Weissman D. (2008). Incorporation of pseudouridine into mRNA yields superior nonimmunogenic vector with increased translational capacity and biological stability. Mol. Ther..

[31] Karikó K., Buckstein M., Ni H., Weissman D. (2005). Suppression of RNA recognition by Toll-like receptors: The impact of nucleoside modification and the evolutionary origin of RNA. Immunity.

[32] Potužník J.F., Cahová H. (2020). It’s the Little Things (in Viral RNA). mBio.

[33] Karikó K., Muramatsu H., Ludwig J., Weissman D. (2011). Generating the optimal mRNA for therapy: HPLC purification eliminates immune activation and improves translation of nucleoside-modified, protein-encoding mRNA. Nucleic Acids Res..

[34] Krušič A., Mencin N., Leban M., Nett E., Perković M., Sahin U., Megušar P., Štrancar A., Sekirnik R. (2025). Reverse-phase chromatography removes double-stranded RNA, fragments, and residual template to decrease immunogenicity and increase cell potency of mRNA and saRNA. Mol. Ther. Nucleic Acids.

[35] Guillerez J., Lopez P.J., Proux F., Launay H., Dreyfus M. (2005). A mutation in T7 RNA polymerase that facilitates promoter clearance. Proc. Natl. Acad. Sci. USA.

[36] Burgin J., Ahamed A., Cummins C., Devraj R., Gueye K., Gupta D., Gupta V., Haseeb M., Ihsan M., Ivanov E. (2023). The European Nucleotide Archive in 2022. Nucleic Acids Res..

[37] Grigoriev I.V., Nordberg H., Shabalov I., Aerts A., Cantor M., Goodstein D., Kuo A., Minovitsky S., Nikitin R., Ohm R.A. (2012). The genome portal of the Department of Energy Joint Genome Institute. Nucleic Acids Res..

[38] Blackwell G.A., Hunt M., Malone K.M., Lima L., Horesh G., Alako B.T.F., Thomson N.R., Iqbal Z. (2021). Exploring bacterial diversity via a curated and searchable snapshot of archived DNA sequences. PLoS Biol..

[39] Edgar R.C., Taylor B., Lin V., Altman T., Barbera P., Meleshko D., Lohr D., Novakovsky G., Buchfink B., Al-Shayeb B. (2022). Petabase-scale sequence alignment catalyses viral discovery. Nature.

[40] Li D., Liu C.-M., Luo R., Sadakane K., Lam T.-W. (2015). MEGAHIT: An ultra-fast single-node solution for large and complex metagenomics assembly via succinct de Bruijn graph. Bioinformatics.

[41] Seemann T. (2014). Prokka: Rapid prokaryotic genome annotation. Bioinformatics.

[42] Buchfink B., Reuter K., Drost H.-G. (2021). Sensitive protein alignments at tree-of-life scale using DIAMOND. Nat. Methods.

[43] Jumper J., Evans R., Pritzel A., Green T., Figurnov M., Ronneberger O., Tunyasuvunakool K., Bates R., Žídek A., Potapenko A. (2021). Highly accurate protein structure prediction with AlphaFold. Nature.

[44] Yang Z., Zeng X., Zhao Y., Chen R. (2023). AlphaFold2 and its applications in the fields of biology and medicine. Signal Transduct. Target. Ther..

[45] Jorgensen E.D., Durbin R.K., Risman S.S., McAllister W.T. (1991). Specific contacts between the bacteriophage T3, T7, and SP6 RNA polymerases and their promoters. J. Biol. Chem..

[46] Filonov G.S., Jaffrey S.R. (2016). RNA Imaging with Dimeric Broccoli in Live Bacterial and Mammalian Cells. Curr. Protoc. Chem. Biol..

[47] Zabierowski S., DeLuca N.A. (2004). Differential cellular requirements for activation of herpes simplex virus type 1 early (tk) and late (gC) promoters by ICP4. J. Virol..

[48] Imburgio D., Rong M., Ma K., McAllister W.T. (2000). Studies of promoter recognition and start site selection by T7 RNA polymerase using a comprehensive collection of promoter variants. Biochemistry.

[49] Curry E., Sedelnikova S., Rafferty J., Hulley M., Pohle M., Muir G., Brown A. (2024). Expanding the RNA polymerase biocatalyst solution space for mRNA manufacture. Biotechnol. J..

[50] Rong M., He B., McAllister W.T., Durbin R.K. (1998). Promoter specificity determinants of T7 RNA polymerase. Proc. Natl. Acad. Sci. USA.

[51] Guzman L.M., Belin D., Carson M.J., Beckwith J. (1995). Tight regulation, modulation, and high-level expression by vectors containing the arabinose PBAD promoter. J. Bacteriol..

[52] Bentley W.E., Mirjalili N., Andersen D.C., Davis R.H., Kompala D.S. (1990). Plasmid-encoded protein: The principal factor in the "metabolic burden" associated with recombinant bacteria. Biotechnol. Bioeng..

[53] Rosano G.L., Ceccarelli E.A. (2014). Recombinant protein expression in Escherichia coli: Advances and challenges. Front. Microbiol..

[54] Deveau H., Garneau J.E., Moineau S. (2010). CRISPR/Cas system and its role in phage-bacteria interactions. Annu. Rev. Microbiol..

[55] Hille F., Richter H., Wong S.P., Bratovič M., Ressel S., Charpentier E. (2018). The Biology of CRISPR-Cas: Backward and Forward. Cell.

[56] Bruder M.R., Aucoin M.G. (2022). Utility of Alternative Promoters for Foreign Gene Expression Using the Baculovirus Expression Vector System. Viruses.

[57] Pohling R. (2015). Chemische Reaktionen in der Wasseranalyse.

[58] Ali S.M., Yosipovitch G. (2013). Skin pH: From basic science to basic skin care. Acta Derm. Venereol..

[59] Bernhardt H.S., Tate W.P. (2012). Primordial soup or vinaigrette: Did the RNA world evolve at acidic pH?. Biol. Direct.

[60] Haley B.J., Chen A., Grim C.J., Clark P., Diaz C.M., Taviani E., Hasan N.A., Sancomb E., Elnemr W.M., Islam M.A. (2012). Vibrio cholerae in an Historically Cholera-Free Country. Environ. Microbiol. Rep..

[61] Kumar P., Libchaber A. (2013). Pressure and temperature dependence of growth and morphology of Escherichia coli: Experiments and stochastic model. Biophys. J..

[62] Chelliserrykattil J., Ellington A.D. (2004). Evolution of a T7 RNA polymerase variant that transcribes 2’-O-methyl RNA. Nat. Biotechnol..

[63] Potapov V., Fu X., Dai N., Corrêa I.R., Tanner N.A., Ong J.L. (2018). Base modifications affecting RNA polymerase and reverse transcriptase fidelity. Nucleic Acids Res..

[64] Padilla R., Sousa R. (2002). A Y639F/H784A T7 RNA polymerase double mutant displays superior properties for synthesizing RNAs with non-canonical NTPs. Nucleic Acids Res..

[65] Zhu B., Hernandez A., Tan M., Wollenhaupt J., Tabor S., Richardson C.C. (2015). Synthesis of 2’-Fluoro RNA by Syn5 RNA polymerase. Nucleic Acids Res..

[66] Tang Q., Zhu S., Hu N., Yin S., Yang Y., Teng Y., Song D., Liu X. (2025). Engineered T7 RNA polymerase reduces dsRNA formation by lowering terminal transferase and RNA-dependent RNA polymerase activities. FEBS J..

[67] Xia H., Yu B., Jiang Y., Cheng R., Lu X., Wu H., Zhu B. (2022). Psychrophilic phage VSW-3 RNA polymerase reduces both terminal and full-length dsRNA byproducts in in vitro transcription. RNA Biol..

