# Supplementary material for: Identification of Phage RNA Polymerases That Minimize Double-Stranded RNA By-Product Formation and Their Characterization via In Vitro Transcription
Source: Microorganisms. 2026 Mar 2;14(3):564. doi: 10.3390/microorganisms14030564 (PMC13029058; doi:10.3390/microorganisms14030564)
Supplement: Supplementary file 1 [file microorganisms-14-00564-s001.zip › microorganisms-4141312-supplementary.pdf]

SUPPLEMENTARY DATA

MANUSCRIPT TITLE

Identification of phage RNA polymerases that minimize dsRNA by-product formation and their characterization via in vitro transcription

AUTHORS

Lilian Göldel, Carsten Bornhövd, Johannes Kabisch, Joseph Heenan, Aron Eiermann, Thomas Brück * and Hagen Richter *

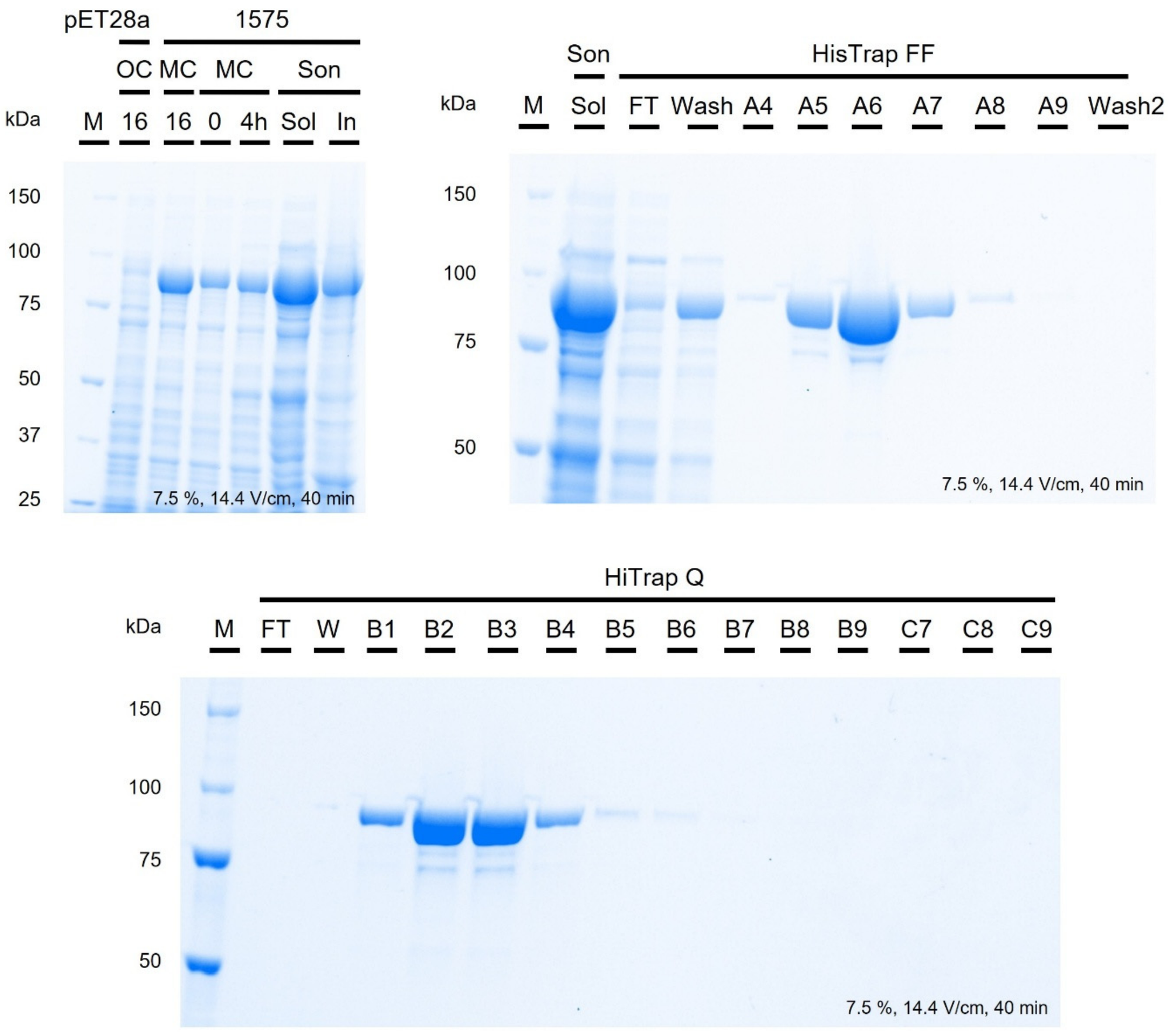

Supplementary Figure S1A: 7.5% SDS gels of cultivation and purification samples of protein No. 1575. M = protein marker, OC = Overnight culture, MC = Main culture, 0, 4, 16 = time of cultivation, Son = Sonication, Sol = Soluble fraction, In = Insoluble fraction, HisTrap FF = Ni^{2+} sepharose column, HiTrap Q = Anion exchange column, FT = Flow-through fraction of the respective column, W = Wash fraction of the respective column, A4–C9 = Elutions of the of the respective column. The soluble fraction after sonication was applied to the HisTrap FF column. Elutions A4–A8 were used as samples for the HiTrap Q column. Elutions B2 and B3 were pooled and transferred into the storage buffer.

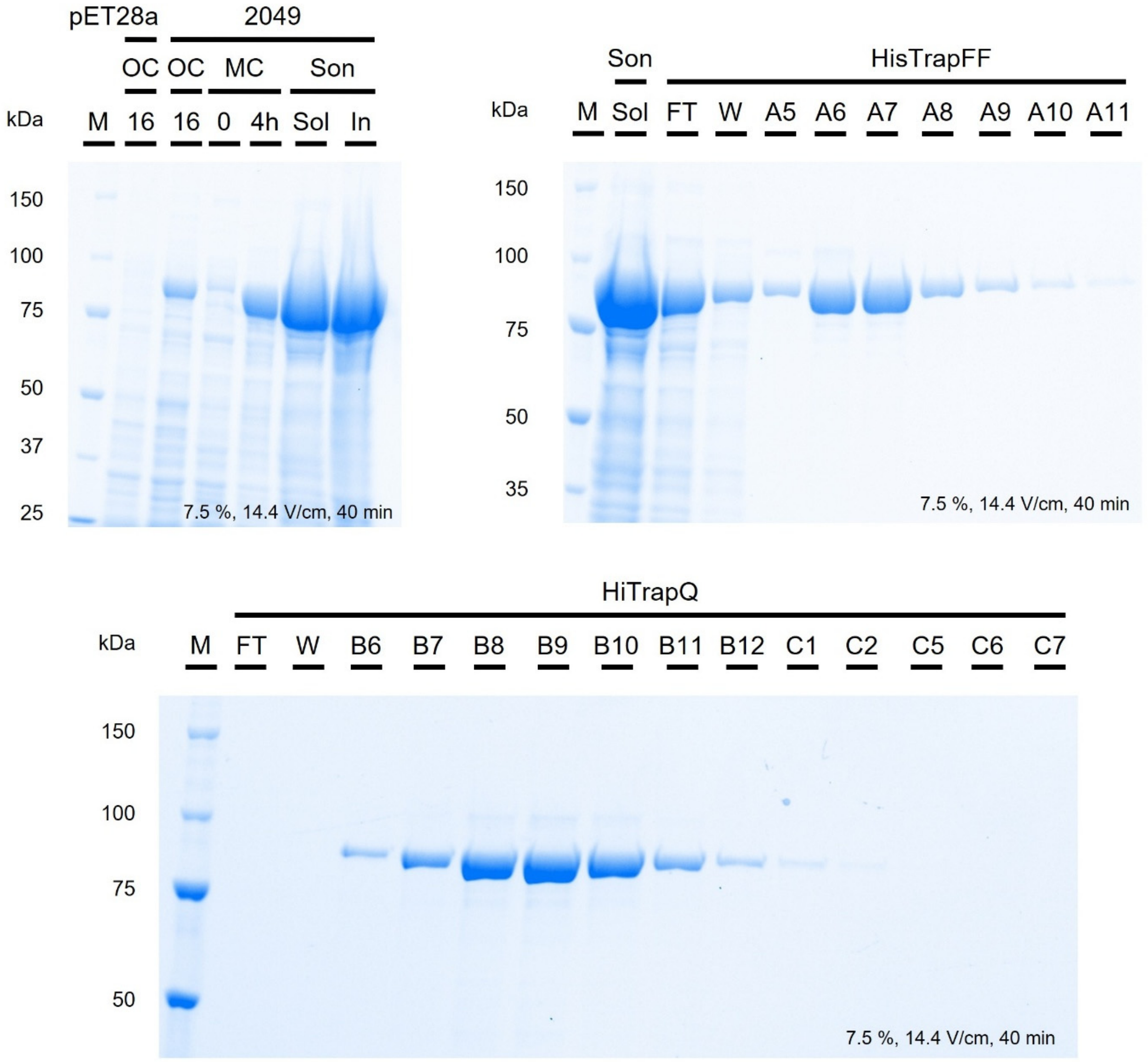

Supplementary Figure S1B: 7.5% SDS gels of cultivation and purification samples of protein No. 2049. M = protein marker, OC = Overnight culture, MC = Main culture, 0, 4, 16 = time of cultivation, Son = Sonication, Sol = Soluble fraction, In = Insoluble fraction, HisTrap FF = Ni^{2+} sepharose column, HiTrap Q = Anion exchange column, FT = Flow-through fraction of the respective column, W = Wash fraction of the respective column, A5–C7 = Elutions of the of the respective column. The soluble fraction after sonication was applied to the HisTrap FF column. Elutions A6–A8 were used as samples for the HiTrap Q column. Elutions B8 and B9 were pooled and transferred into the storage buffer.

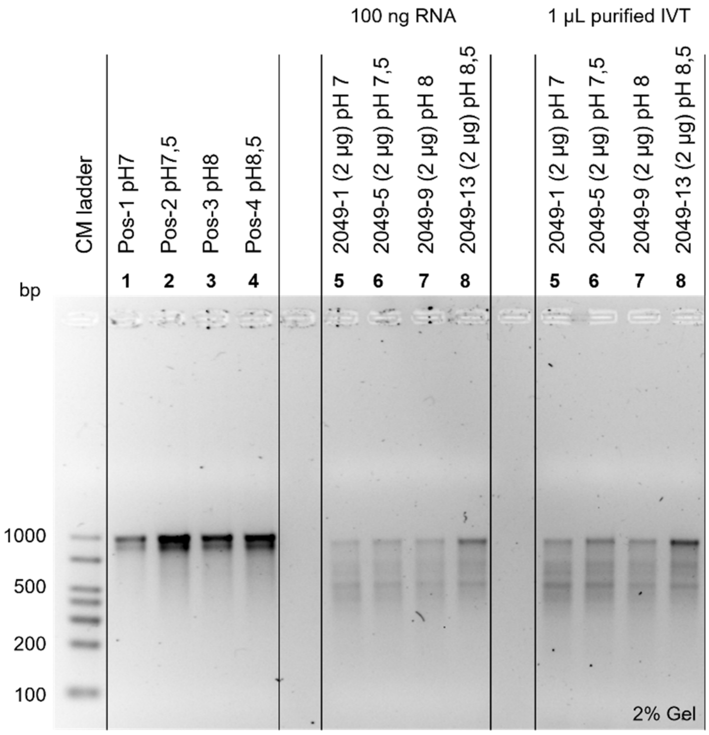

Supplementary Figure S2: 2% agarose gel of purified mRNA samples of IVTs performed with T7 RNA polymerase, protein No. 2049 on a DNA template with the T7 or the 2049 promoter and the gene for GFP. The ladder is Century Plus marker. N = 1

Potential promoter sequence	Position of sequence in genome assembly	Gene downstream of sequence
TATTTAGCTGACACTATAAGAGAA	2,284	Hypothetical protein
TATTTACCGGACACTATAGGAGAG	6,041	Bac.phage 1.1 protein
GATTTACCGGACACTATAGAAGAG	8,701	Hypothetical protein
CATTTTGCCGACACTATAGAAGAC	9,431	Hypothetical protein
GATTTACTGGACACTATAGAAGGA	12,873	Hypothetical protein
AATTTACTGGACACTATAGAACAA	13,336	Hypothetical protein
TATTTACTGGACACTATAGAAGAG	14,923	Hypothetical protein
GATTTAGGTGACACTATAGAACAA	18,099	Nucleotide kinase gp1.7
TATTTACTGGACACTATAGAAGGG	23,035	Minor capsid protein
ACATTAGGTGACACTATAGAAGTA	30,000	Peptidoglycan hydolase gp36
GCAATACTGGACACTATAGAAGGA	36,271	Hypothetical protein
TATTTACTGGACACTATAGGAGGA	38,376	Hypothetical protein
GATTTACTGGACACTATAGAAGAG	40,718	Hypothetical protein

Supplementary Figure S3A: List of 14 potential promoter sequences for RNAP variant No. 2049 and their genetic context.

No.	Promoter sequence
1	TATTTAGGTGACACTATAGAAGAG
2	TAATTAGAGACCACTATAGAAGAA
3	TATTTACTGGACACTATAGAAGAA
4	TATTTACTGGACACTATAGAAGGA
5	TATTTACTGGACACTATAGGAGAA
6	TATTTACTGGACACTATAGGAGGA
7	TATTTACTGGACACTATAGGGGAA
8	TATTTACTGGACACTATAGGGGGA

Supplementary Figure S3B: List of final potential promoter sequences for RNAP variant No. 2049 selected for further investigation.

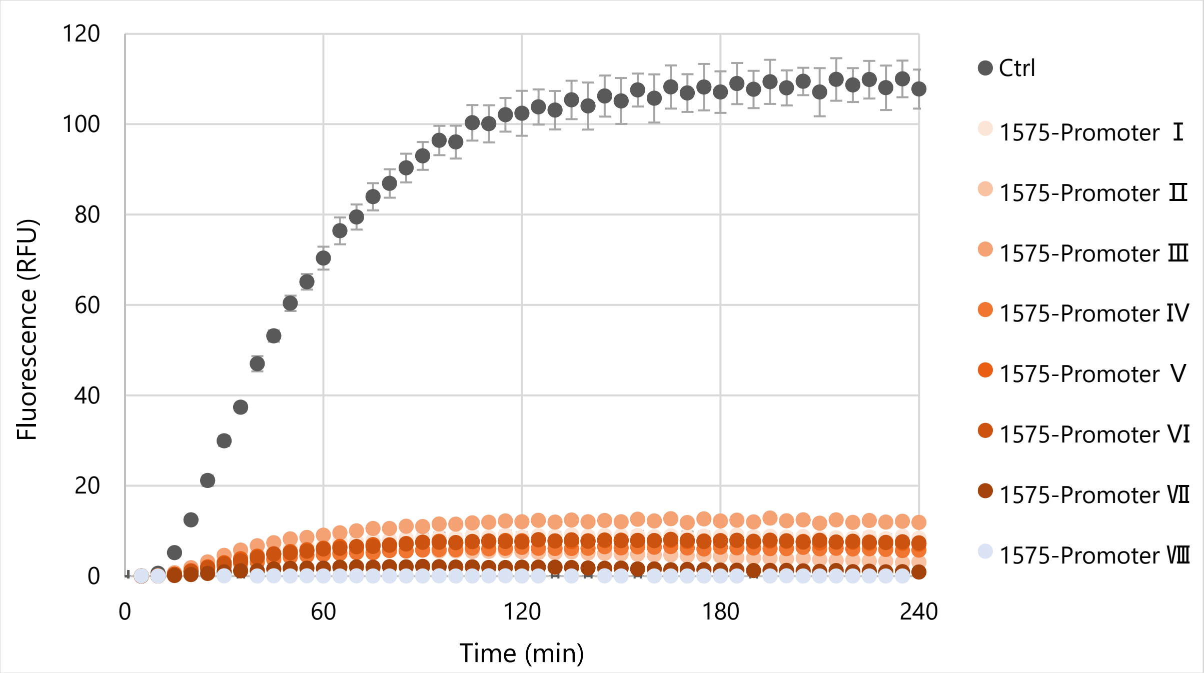

Supplementary Figure S4A: Activity of RNAP No. 1575 using different promoters 1–8 at pH 7.5 for 240 min measured in fluorescence (RFU). N = 3

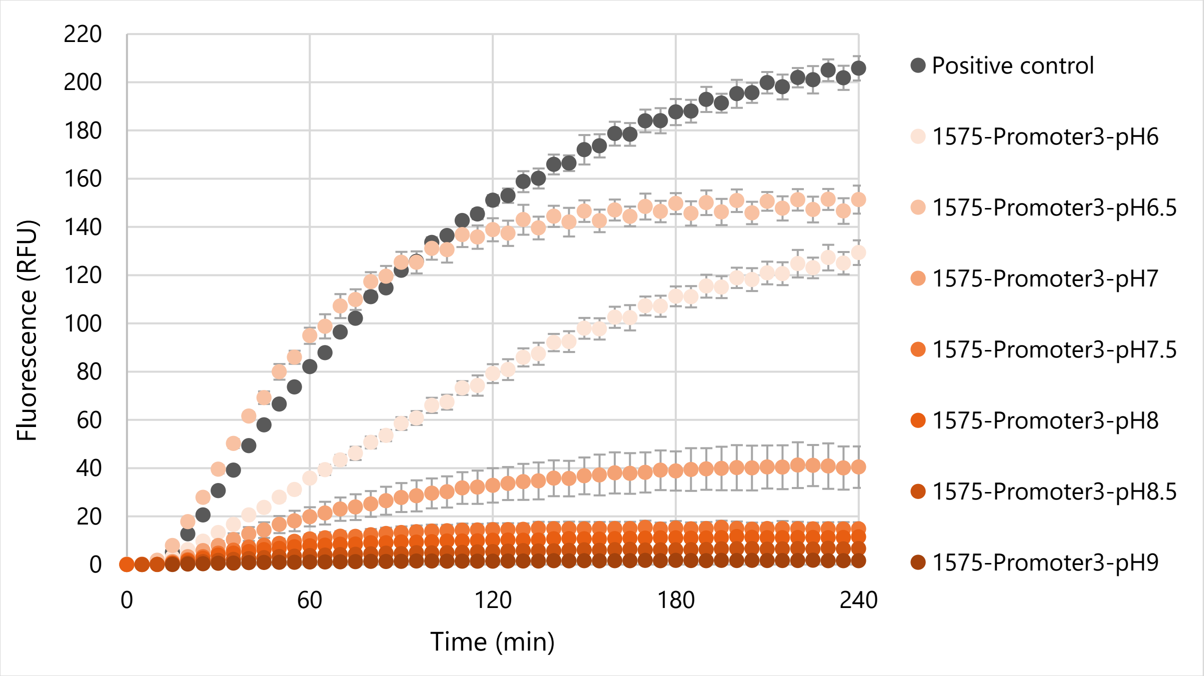

Supplementary Figure S4B: Activity of RNAP No. 1575 using promoter 3 at various pH values for 240 min measured in fluorescence (RFU). N = 3

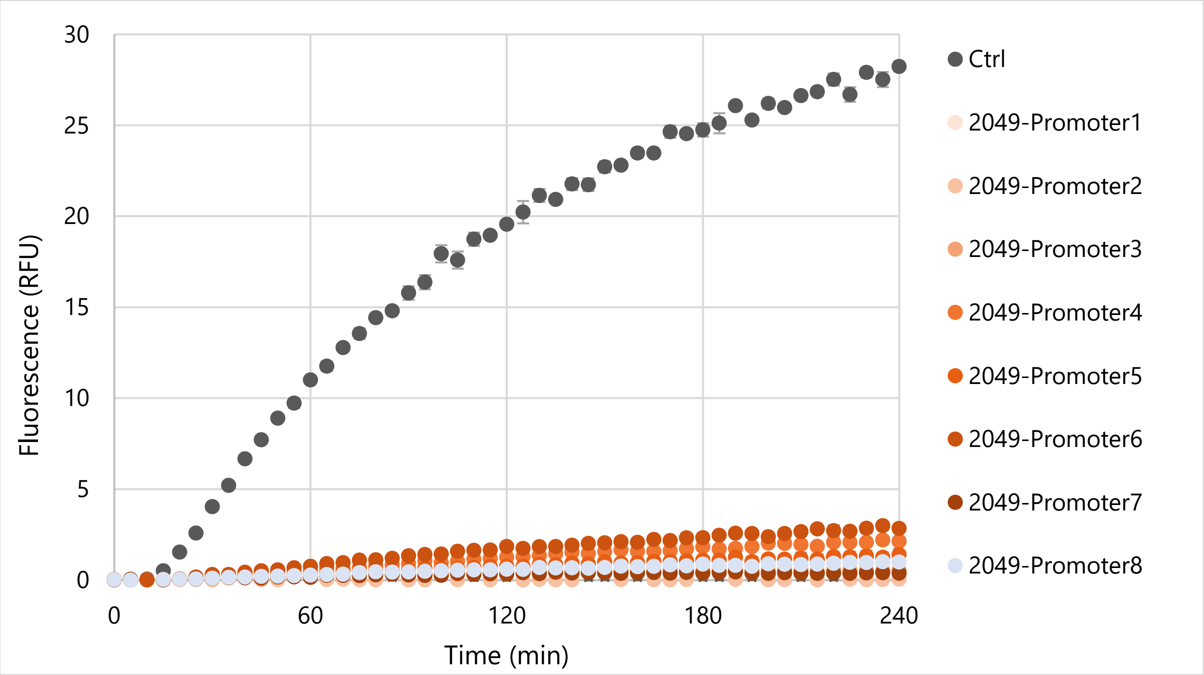

Supplementary Figure S4C: Activity of RNAP No. 2049 using different promoters 1–8 at pH 7.5 for 240 min measured in fluorescence (RFU). N = 3

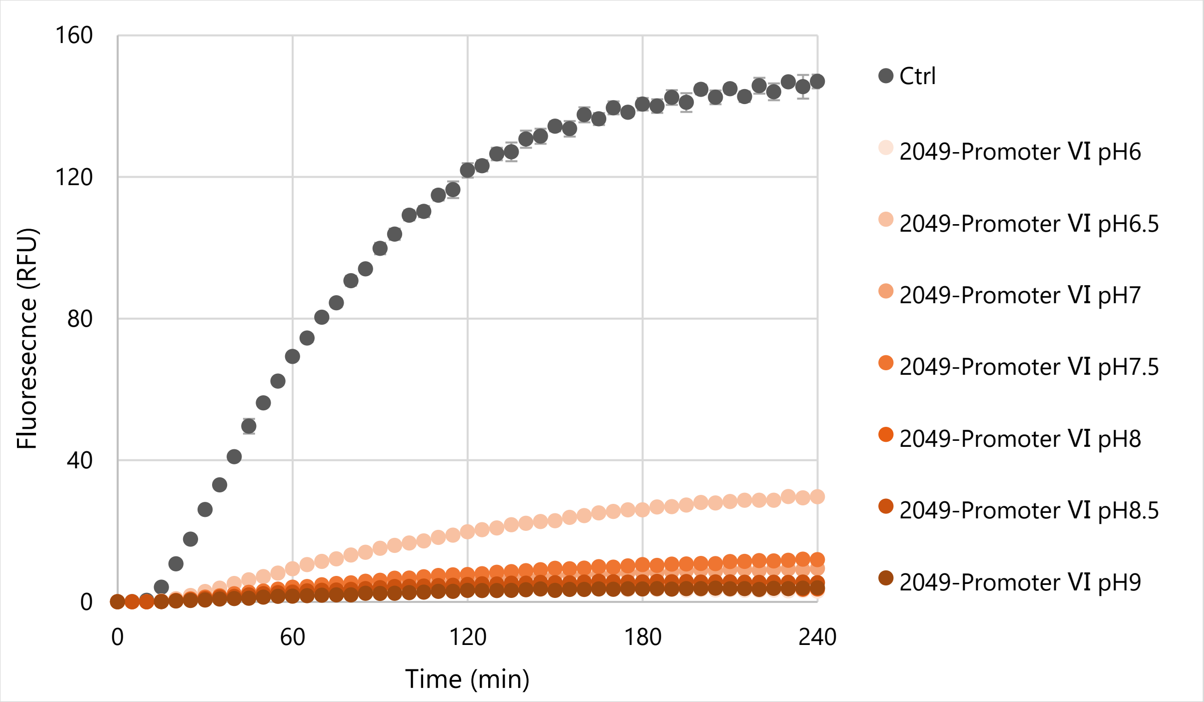

Supplementary Figure S4D: Activity of RNAP No. 2049 using promoter 6 at various pH values for 240 min measured in fluorescence (RFU). N = 3

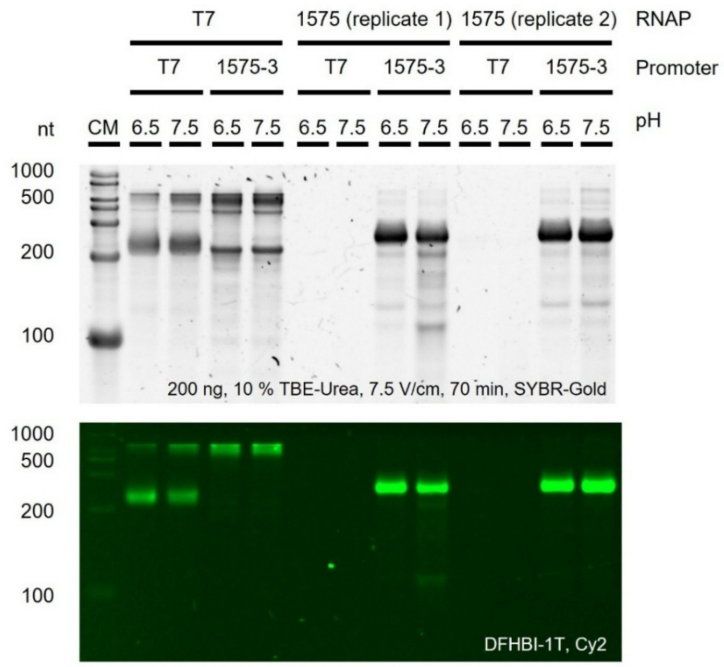

Supplementary Figure S4E: 10 % TBE-Urea gel of purified mRNA samples (200 ng) of IVTs performed at pH 6.5 or 7.5 with T7 RNA polymerase or protein No. 1575 on a DNA template with the T7 or the 1575-3 promoter and the gene for 2xdBroccoli. The ladder is Century Plus marker. First, the Gel was stained with DFHBI-1T (40 µM) for detection of 2xdBroccoli and subsequently with SYBR Gold for detection of total RNA. N = 1

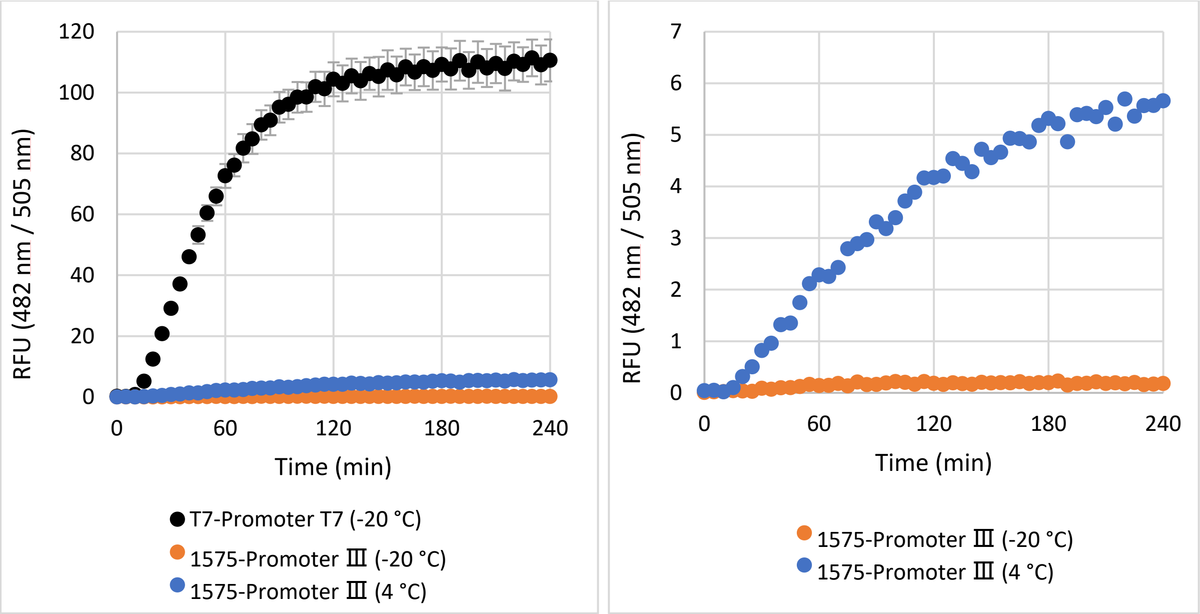

Supplementary Figure S4F: RNAP No. 1575 activity using promoter 3 at pH 7.5 for 240 min measured in fluorescence (RFU). The RNA polymerases applied were stored 27 days at the respective temperature before used in fluorescent *in vitro* transcription assays (IVTs). The right panel depicts the same data as the left panel, but without the data obtained for T7 RNAP to highlight the difference in RFU of IVTs with No. 1575 stored at different temperatures (−20 °C or 4 °C). N = 3 for T7 RNAP and No. 1575 stored at −20 °C, N = 1 for No. 1575 stored at 4 °C

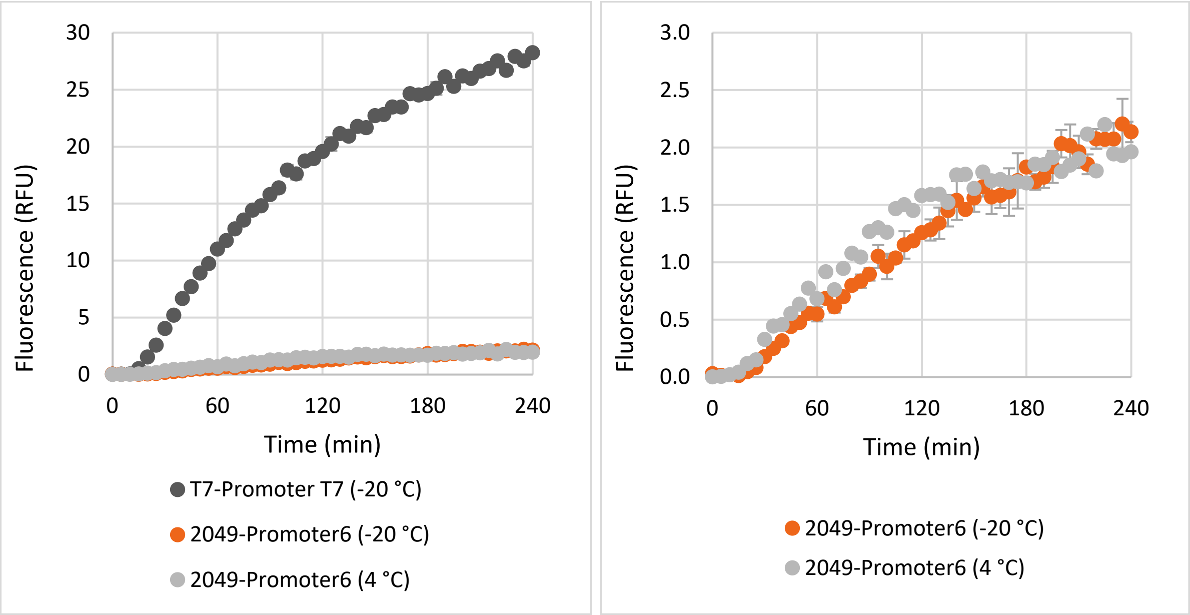

Supplementary Figure S4G: RNAP No. 2049 activity using promoter 6 at pH 7.5 for 240 min measured in fluorescence (RFU). The RNA polymerases applied were stored 27 days at the respective temperature before used in fluorescent *in vitro* transcription assays (IVTs). The right panel depicts the same data as the left panel, but without the data obtained for T7 RNAP to highlight the difference in RFU of IVTs with No. 2049 stored at different temperatures (−20 °C or 4 °C). N = 3 for T7 RNAP and No. 2049 stored at −20 °C, N= 1 for No. 2049 stored at 4 °C

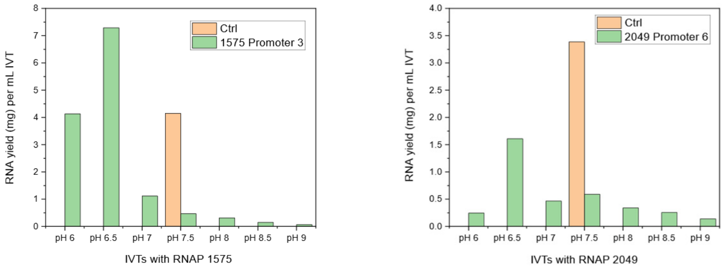

Supplementary Figure S5A: RNA yield of 2xdBroccoli mRNA produced by T7 RNAP (ctrl), RNAP No. 1575 and RNAP No. 2049 at 37 °C. N = 3

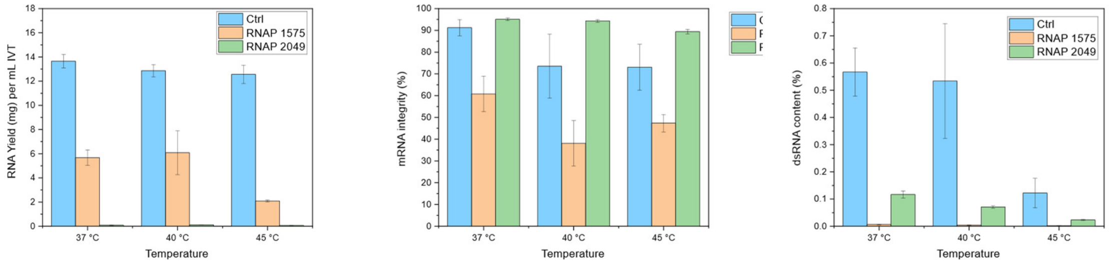

Supplementary Figure S5B: RNA yield, dsRNA content and mRNA integrity of GFP mRNA produced by T7 RNAP (ctrl), RNAP No. 1575 and RNAP No. 2049 at 37, 40 and 45 °C. N = 3

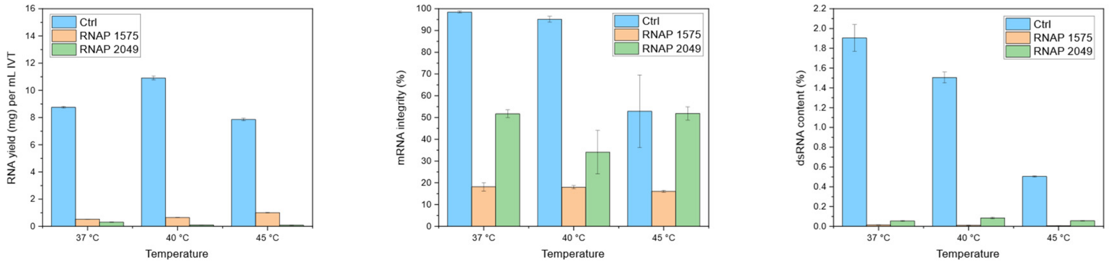

Supplementary Figure S5C: RNA yield, dsRNA content and mRNA integrity of FLuc mRNA produced by T7 RNAP (ctrl), RNAP No. 1575 and RNAP No. 2049 at 37, 40 and 45 °C. N = 3

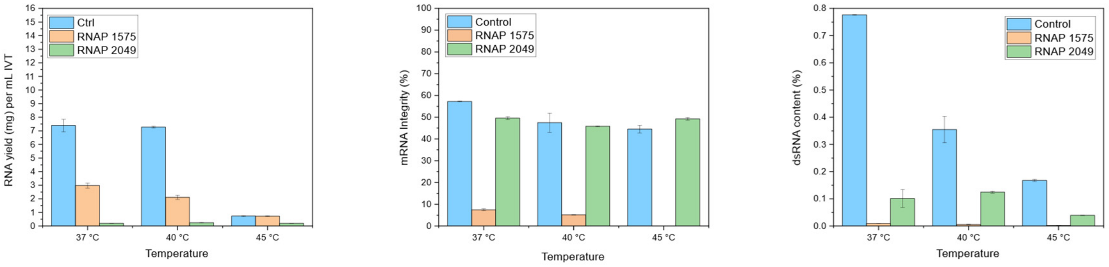

Supplementary Figure S5D: RNA yield, dsRNA content and mRNA integrity of Cas9 mRNA produced by T7 RNAP (ctrl), RNAP No. 1575 and RNAP No. 2049 at 37, 40 and 45 °C. N = 3

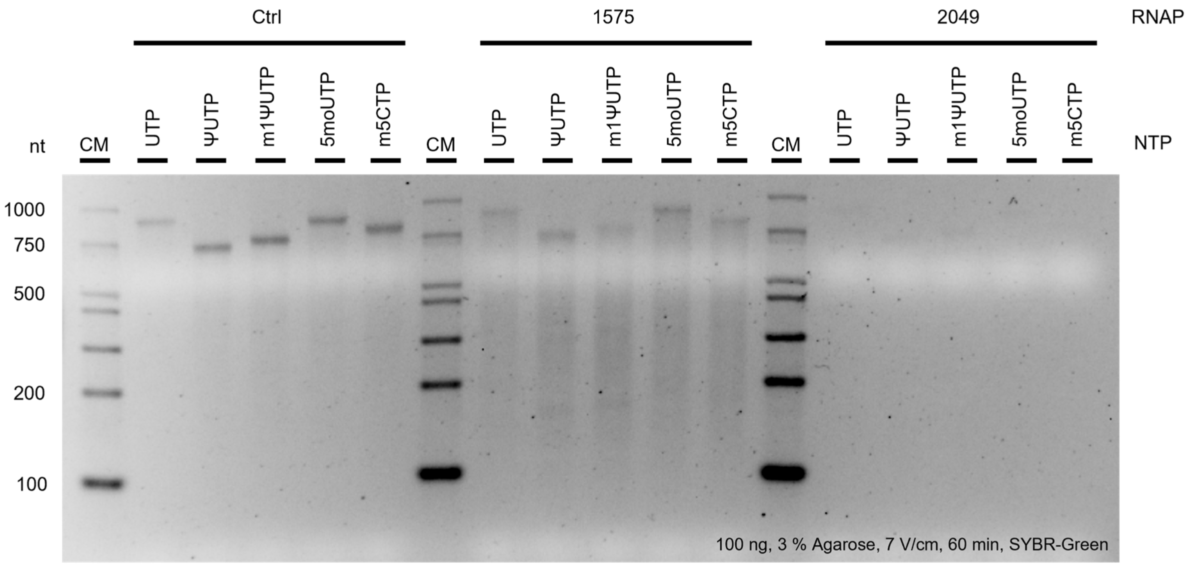

Supplementary Figure S5E: 3 % agarose gel of GFP mRNA produced by T7 RNAP (ctrl), RNAP No. 1575 with Pseudo-UTP, N1-methylpseudo-UTP, 5-methoxy-UTP, 5-methyl-CTP. N = 1
